# Integration of postpartum healthcare services for HIV-infected women and their infants in South Africa: A randomised controlled trial

**DOI:** 10.1371/journal.pmed.1002547

**Published:** 2018-03-30

**Authors:** Landon Myer, Tamsin K. Phillips, Allison Zerbe, Kirsty Brittain, Maia Lesosky, Nei-Yuan Hsiao, Robert H. Remien, Claude A. Mellins, James A. McIntyre, Elaine J. Abrams

**Affiliations:** 1 Division of Epidemiology and Biostatistics, School of Public Health and Family Medicine, University of Cape Town, Cape Town, South Africa; 2 Centre for Infectious Diseases Epidemiology and Research, School of Public Health and Family Medicine, University of Cape Town, Cape Town, South Africa; 3 ICAP at Columbia University, Mailman School of Public Health, Columbia University, New York, New York, United States of America; 4 National Health Laboratory Service, Cape Town, South Africa; 5 Division of Medical Virology, University of Cape Town, Cape Town, South Africa; 6 HIV Center for Clinical and Behavioral Studies, New York State Psychiatric Institute, New York, New York, United States of America; 7 Anova Health Institute, Johannesburg, South Africa; 8 College of Physicians and Surgeons, Columbia University, New York, New York, United States of America; 9 Department of Epidemiology, Mailman School of Public Health, Columbia University, New York, New York, United States of America; University of California, San Francisco, UNITED STATES

## Abstract

**Background:**

As the number of HIV-infected women initiating lifelong antiretroviral therapy (ART) during pregnancy increases globally, concerns have emerged regarding low levels of retention in HIV services and suboptimal adherence to ART during the postpartum period. We examined the impact of integrating postpartum ART for HIV+ mothers alongside infant follow-up within maternal and child health (MCH) services in Cape Town, South Africa.

**Methods and findings:**

We conducted a randomised trial among HIV+ postpartum women aged ≥18 years who initiated ART during pregnancy in the local antenatal care clinic and were breastfeeding when screened before 6 weeks postpartum. We compared an integrated postnatal service among mothers and their infants (the MCH-ART intervention) to the local standard of care (control)—immediate postnatal referral of HIV+ women on ART to general adult ART services and their infants to separate routine infant follow-up. Evaluation data were collected through medical records and trial measurement visits scheduled and located separately from healthcare services involved in either arm. The primary trial outcome was a composite endpoint of women’s retention in ART care and viral suppression (VS) (viral load < 50 copies/ml) at 12 months postpartum; secondary outcomes included duration of any and exclusive breastfeeding, mother-to-child HIV transmission, and infant mortality. Between 5 June 2013 and 10 December 2014, a total of 471 mother–infant pairs were enrolled and randomised (mean age, 28.6 years; 18% nulliparous; 57% newly diagnosed with HIV in pregnancy; median duration of ART use at randomisation, 18 weeks). Among 411 women (87%) with primary endpoint data available, 77% of women (*n =* 155) randomised to the MCH-ART intervention achieved the primary composite outcome of retention in ART services with VS at 12 months postpartum, compared to 56% of women (*n =* 117) randomised to the control arm (absolute risk difference, 0.21; 95% CI: 0.12–0.30; *p <* 0.001). The findings for improved retention in care and VS among women in the MCH-ART intervention arm were consistent across subgroups of participants according to demographic and clinical characteristics. The median durations of any breastfeeding and exclusive breastfeeding were longer in women randomised to the intervention versus control arm (6.9 versus 3.0 months, *p =* 0.006, and 3.0 versus 1.4 months, *p <* 0.001, respectively). For the infants, overall HIV-free survival through 12 months of age was 97%: mother-to-child HIV transmission was 1.2% overall (*n =* 4 and *n =* 1 transmissions in the intervention and control arms, respectively), and infant mortality was 1.9% (*n =* 6 and *n =* 3 deaths in the intervention and control arms, respectively), and these outcomes were similar by trial arm. Interpretation of these findings should be qualified by the location of this study in a single urban area as well as the self-reported nature of breastfeeding outcomes.

**Conclusions:**

In this study, we found that integrating ART services into the MCH platform during the postnatal period was a simple and effective intervention, and this should be considered for improving maternal and child outcomes in the context of HIV.

**Trial registration:**

ClinicalTrials.gov NCT01933477.

## Abstract 2

In a randomised controlled trial, Landon Myer and colleagues study the effect of an integrated maternal and child health intervention on provision of HIV care.

## Author summary

**Why was this study done?:**

- More than 1 million HIV-infected women of reproductive age become pregnant globally each year, and World Health Organization policies call for initiation of lifelong antiretroviral therapy (ART) in all HIV-infected pregnant and breastfeeding women.
- However, keeping HIV-infected women retained in care, adherent to ART, and with HIV viral suppression is a major public health challenge, particularly postpartum.
- Integration of ART services into existing maternal and child health (MCH) programmes is one intervention approach that has been widely discussed to address this challenge, but this approach has not been rigorously evaluated.

**What did the researchers do and find?:**

- Working in Cape Town, South Africa, we recruited immediately postpartum 471 HIV-infected, breastfeeding women who had initiated ART during pregnancy.
- We randomised mother–infant pairs to either referral to general adult ART clinics, with their children referred to child health clinics (the local standard of care, which acted as a control condition), or keeping mothers and infants in care together in an MCH-focused ART service (the intervention).
- We followed mother–infant pairs through 12 months postpartum with regular study visits that were scheduled and conducted independent of routine services in either the intervention or control arms; batched viral load testing was conducted at these study visits, separate from routine care.
- We found that women randomised to the integrated care intervention had higher levels of retention in care and viral suppression through 12 months postpartum. This difference was observed in all subgroups of women, including younger and older women, those newly diagnosed with HIV, those with lower CD4 cell counts, and those with longer durations of ART use before delivery.
- Women in the integrated care intervention also reported breastfeeding their infants for longer periods. Levels of infant mortality and mother-to-child HIV transmission were low and similar between the intervention and control arms.

**What do these findings mean?:**

- Integrating MCH and ART services appears effective in keeping HIV-infected mothers retained in care and virally suppressed.
- Integrated services may also contribute to longer durations of breastfeeding, and thus contribute to child health.
- These data should be interpreted carefully, as they come from a single part of South Africa, and further research is needed to examine the generalisability of the findings to other settings.

## Introduction

The past decade has witnessed a paradigm shift in the science of and services for prevention of mother-to-child HIV transmission (PMTCT) [1,2]. Lifelong triple-drug antiretroviral therapy (ART) for all women living with HIV regardless of disease status (per the World Health Organization’s Option B+ approach) has emerged as the standard for reducing vertical HIV transmission and promoting maternal health [3]. With this approach, there have been substantial increases globally in ART use by HIV+ pregnant and postpartum women, with consequent reductions in new paediatric HIV infections, providing a potent example of the synergies of ART use for HIV treatment and prevention [4].

Alongside the successes of universal ART for pregnant and postpartum women, multiple studies from around the world have raised major concerns regarding low levels of retention in HIV services and adherence to ART medications in this population [5–8]. These findings appear especially marked in the postpartum period and contribute directly to failures in maintaining viral suppression (VS) [8,9]. This limits the benefits of ART, and, in turn, there is an urgent need to help HIV+ women to remain engaged in ART services over the long term. Moreover, with new WHO guidelines recommending that HIV+ mothers breastfeed for at least 12 months postpartum [10], the ability of health services to support mothers on ART has direct implications for the health of their HIV-exposed infants. Yet there have been few rigorous evaluations of interventions to promote postpartum engagement in ART services, and existing evaluations have been limited in their duration of follow-up [11].

Related to this, there has been a call for integrating HIV care and treatment services into the broader platform of primary healthcare, particularly in the context of PMTCT [12]. Integration of ART into antenatal care (ANC) services has proven critical to the successful delivery of ART to pregnant women under Option B+ across sub-Saharan Africa [13]. However, there has been little consideration given to the integration of health services during the postpartum period, when HIV+ women’s retention and adherence may be under greatest threat. In many settings, women initiating ART as part of ANC are referred immediately post-delivery to general adult ART services [14,15]. This change in the location of care and the separation of maternal and infant services has been a point of loss to follow-up, and there may be a major missed opportunity for linking ART services within the broader maternal and child health (MCH) care platform. To address this gap, we examined the impact of integrating postpartum ART for HIV+ mothers and infant follow-up within MCH services on maternal retention in care and VS through 12 months postpartum in Cape Town, South Africa.

## Methods

### Design and setting

We conducted a parallel-arm randomised trial comparing an integrated postnatal care service within the MCH setting for HIV+ mothers and their infants (the MCH-ART intervention) to the local standard of care (SOC, control)—immediate postnatal referral of HIV+ women on ART to general adult ART services and their infants to routine infant follow-up. The trial took place in a subdistrict of Cape Town as described previously [16]. Within the subdistrict, the study was based at a large, primary-level antenatal and obstetric facility located in a community of approximately 300,000 where levels of both poverty and HIV infection are high; referral was to one of a number of primary care services in the subdistrict [17]. The study was registered on ClinicalTrials.gov (NCT01933477); final registration was approved 3 months after the start of enrolment due to an administrative error. The study protocol is provided as S1 Text. The study protocol was approved by the Human Research Ethics Committee of the University of Cape Town Faculty of Health Sciences, as well as by the Institutional Review Board of Columbia University Medical Center.

### Routine PMTCT and ART services

In this setting, routine ANC is provided in the MCH clinic and includes PMTCT services with nurse-midwives initiating women on ART and providing follow-up with support from counsellors. ART eligibility is based on local public sector guidelines. For women making their first ANC visit before July 2013, eligibility was based on CD4 cell count ≤ 350 cells/μl or WHO stage III/IV disease, per WHO 2010 recommendation Option A. From July 2013, all HIV+ pregnant women were ART eligible (WHO 2013 recommendation Option B+) [18]. All women initiated the local first-line ART regimen of tenofovir (300 mg) + emtricitabine (200 mg)/lamivudine (300 mg) + efavirenz (600 mg) provided as a fixed-dose combination pill taken once daily. For all women in the study, ART initiation, all clinical care, and infant follow-up was provided by government health services based on public sector policies. Routine infant HIV diagnostic testing in this setting, based on nucleic acid amplification tests, was carried out at 6–10 weeks of age.

### Participants and eligibility

Participants were HIV+ women and their infants (regardless of HIV infection status) recruited immediately postpartum during routine clinical follow-up visits. As part of a larger implementation science study, all HIV+ women seeking ANC who were eligible for enrolment were interviewed and followed from their first ANC visit. To be eligible for the trial, women were required to be at least 18 years of age and less than 6 weeks postpartum, to have initiated ART during the recently completed pregnancy (regardless of previous antiretroviral [ARV] exposure or evidence of VS), and to be breastfeeding their infants at the time of screening.

### Randomisation

After providing written informed consent, eligible mother–infant pairs were allocated 1:1 to the intervention or control arm via computer-generated block randomisation with dynamically permuted block sizes from 2 to 8. Allocations were via opaque sealed envelopes kept securely and accessed by the study coordinator. The study coordinator communicated the random allocation to nurse-midwives in the MCH clinic via a standardised letter.

### MCH-ART intervention

Women randomised to the intervention continued to receive postnatal care in the MCH clinic for the duration of breastfeeding, with care provided to their HIV-exposed infants in the same service. As part of this, nurse-midwives provided ongoing HIV-specific services (including ART and routine infant HIV diagnostic testing) in addition to general care (including contraception for mothers and routine infant care such as anthropometry and vaccinations). At each postnatal visit, nurse-midwives asked mothers about their infant feeding; when mothers reported having stopped breastfeeding, nurse-midwives conducted additional infant HIV diagnostic testing per local guidelines and referred mother–infant pairs out of the MCH clinic, although study-specific follow-up persisted regardless of the site of care.

### SOC control

Women and their infants randomised to the control arm followed the local SOC, with referral out of the MCH clinic—to general adult ART services for the mother and “well baby” (child health) services for the infant—by nurse-midwives as soon as possible postpartum, typically at their first postnatal visit.

### Referrals

For both the intervention and control arms, referrals out of the MCH clinic followed routine local practice. Women were counselled on ART adherence, infant feeding and care including HIV testing, and related aspects of maternal and infant health. They were provided with a referral letter to attend the ART clinic of their choosing (typically based on area of residence) and dispensed a 1-month supply of ART. Mothers were advised to take infants as soon as possible to their nearest routine child health services, which incorporated care for HIV-exposed infants.

### Counselling and patient support

Under both the intervention and control conditions, ART counselling was provided by the routine public sector ART counsellors based either in the MCH clinic (intervention) or general adult ART services (control). These counsellors undergo standardised training and supervision; throughout the study, counselling messages and frequency were based on local standards without adaptation in either the intervention or control arm.

### Missed clinic visits

Procedures for tracing women who did not attend a scheduled clinic appointment in either the intervention or control arm were based on local public sector norms: as feasible according to available information and clinic workloads, clinic personnel attempted to contact patients who missed a clinic visit via telephone and/or home visit.

### Sources of data

Evaluation data were collected through a series of trial measurement visits scheduled and located separately from the public sector healthcare services involved in either arm of the trial. Visits took place at 6 weeks and then 3, 6, 9, and 12 months postpartum. For attendance at each trial measurement visit, women received R 150 (approximately US$12) in a combination of food vouchers and/or child-related consumables (such as diapers or clothing); no reimbursement was provided for attendance at routine healthcare visits in either the intervention or control arm.

At the start of each measurement visit, participating mothers were instructed to not disclose to study personnel where they were receiving HIV care, and questionnaires did not investigate this. Mothers provided 5 ml of venous blood at each study visit for batched HIV RNA viral load (VL) testing by the South African National Health Laboratory Services (NHLS) using the Abbott RealTime HIV-1 assay (Abbott Laboratories, Abbott Park, Illinois, US); this testing was conducted independently from routine VL monitoring for patients on ART. At the 12-month study visit, infants underwent phlebotomy for HIV PCR testing at NHLS using the Roche COBAS AmpliPrep/COBAS TaqMan HIV-1 assay (Roche Diagnostics, Branchburg, New Jersey, US). In the event of missed trial measurement visits, participants were traced by a study fieldworker working independently of routine healthcare services.

Additional data on maternal and infant use of healthcare services were drawn from routinely collected public sector medical records, abstracted for all participants at the end of the study period. Data sources for this were identical across facilities involved in both arms of the trial and included medical record review at all care facilities (both from folders and electronic medical records, when available, and including infants’ Road to Health Booklets), provincial electronic pharmacy dispensing records, and centralised NHLS databases of laboratory tests.

### Outcomes

The primary trial outcome was a composite endpoint of women’s retention in ART care and VS (VL < 50 copies/ml based on VL testing at trial measurement visit) at 12 months postpartum. Retention in care at 12 months postpartum was measured using routinely collected medical records and defined as evidence of an HIV-related clinical contact from the period 9–18 months postpartum. Data used to define a clinical contact came from routinely collected records of ARV dispensing, HIV-related laboratory testing, and clinical care visits. In sensitivity analyses, we examined (i) alternate window periods used to define 12-month retention and (ii) whether the source of retention data influenced trial findings. Secondary outcomes included the separate components of the primary outcome, time to loss of VS, duration of any breastfeeding and exclusive breastfeeding (EBF), infant engagement in appropriate care including HIV testing and use of ARV prophylaxis, maternal family planning use, mother-to-child HIV transmission, and infant mortality. Because outcome data on retention in ART care came from medical records and did not require separate study follow-up, this outcome was available for all participants (regardless of completion of trial measurement visits and availability of VL outcome data) and is presented for all women enrolled as well as restricted to women who also had VL outcome data.

### Statistical analysis

The trial sample size was based on a superiority comparison using 90% power and a 2-sided alpha at 0.05. With 1:1 randomisation, we aimed to detect an absolute difference in the primary outcome between trial arms of at least 15%, based on an estimated 90% and 75% of women retained in care and virally suppressed in the MCH-ART and SOC arms, respectively. With an estimated 20% loss to follow-up from the trial by 12 months postpartum, we calculated that approximately 390 women would need to be randomised. When local PMTCT policies changed from Option A to Option B+ in July 2013, we revised the target sample size to be based on 390 women initiating ART under Option B+ but continued to follow up women enrolled in the trial who initiated ART under Option A.

Analyses used Stata version 13.0 (StataCorp, College Station, Texas, US) and R (R Foundation for Statistical Computing, Vienna, Austria). Throughout, results are presented both for all women enrolled and those initiating ART under Option B+; because findings did not differ between these two groups, we focus primarily on the total randomised population. Unless otherwise specified, all analyses are by intention to treat. Variables were described using means (with standard deviations), medians (with interquartile ranges), and proportions (with 95% confidence intervals). We used rank-sum and chi-squared (replaced in the case of sparse data by Fisher’s exact) tests for bivariate analyses; all statistical tests are 2-sided at α = 0.05. Product-limit methods were used for time-to-event analyses, with the log-rank test used to compare survival times. VS was defined as VL < 50 copies/ml for the primary outcome, with additional analyses for VL < 1,000 copies/ml based on international guidelines. Additive binomial regression models [19] were used to examine the effect of trial arm on the primary outcome after adjusting for participant clinical and demographic characteristics; results are presented as absolute risk differences with 95% CIs. Covariates for consideration in these models were selected based on a priori plausibility in predicting either component of the composite endpoint, while not being plausible causal intermediates of any intervention effect; we included in the final model those covariates whose inclusion altered the association of interest (the main trial effect) or which appeared independently associated with the outcome. To examine the potential influence of missing VL outcome data, multiple imputation via chained equations was used to create 25 datasets with complete composite endpoint data for use in sensitivity analyses [20]. Estimates and CIs for imputed data analyses were pooled using Rubin’s rules [21].

## Results

**Fig 1:**
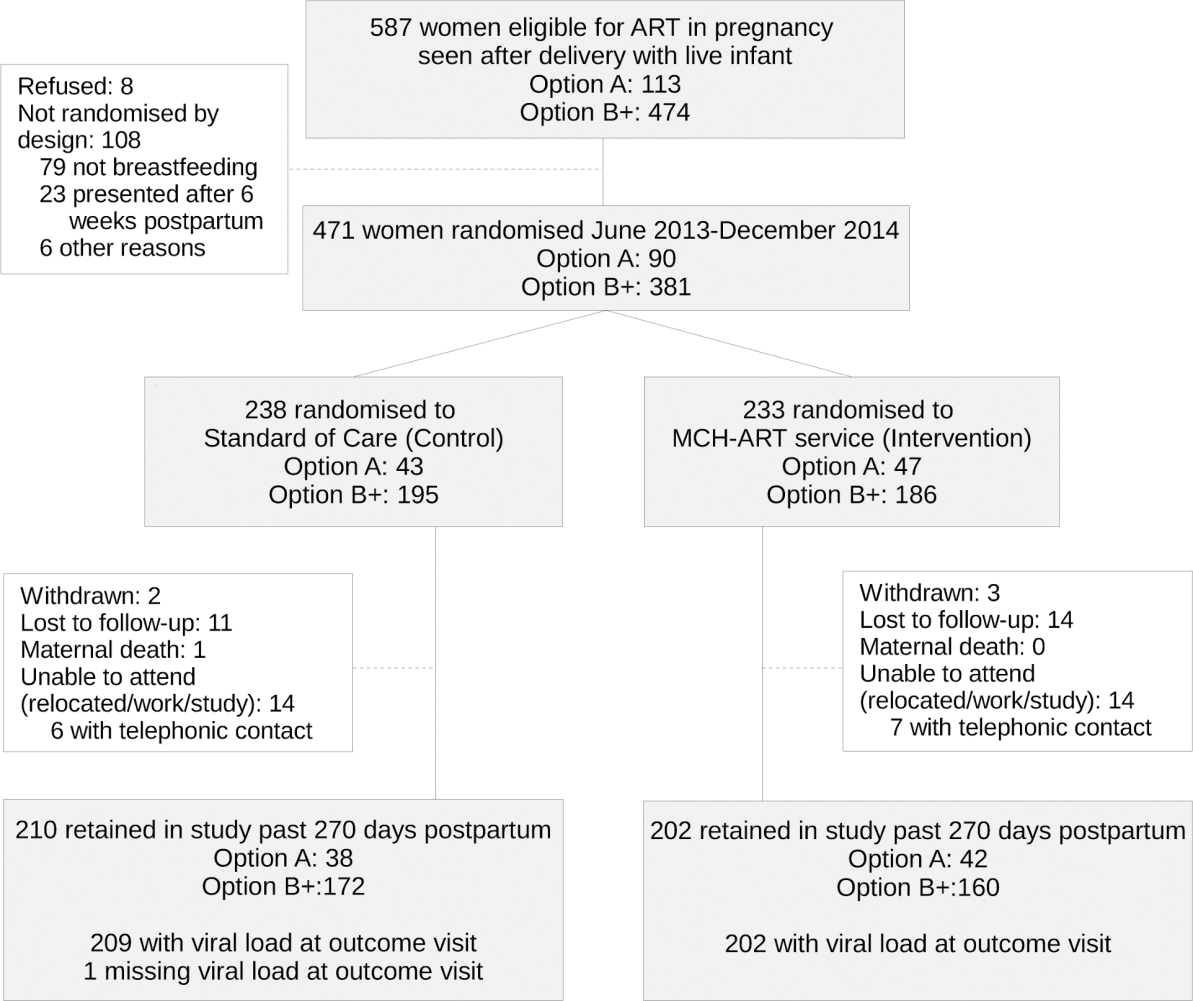
Study enrolment, randomisation, and follow-up.

Between 5 June 2013 and 10 December 2014, a total of 587 women were screened postpartum for inclusion in the trial, of whom 471 were enrolled and randomised (Fig 1). The main reasons for ineligibility of postpartum mother–infant pairs were the mother’s decision not to breastfeed (13%) or presentation outside the 6-week eligibility window (4%); 8 refusals were noted (1%). Of the 471 breastfeeding mother–infant pairs randomised into the trial at a median of 5 days postpartum (IQR, 4–7), 381 (81%) initiated ART under Option B+.

### Characteristics at randomisation

Table 1 shows pre-ART and immediate postpartum characteristics by allocation of women to trial arms, overall and restricted to women enrolled under Option B+. At the first ANC visit, the mean age was 28.6 years, 18% of women (*n =* 87) were nulliparous, and the median gestational age at the start of ANC was 21 weeks (IQR, 16–27). The median CD4 cell count was 354 cells/μl (IQR, 248–517), and the median log_10_ VL was 4.0 copies/ml (IQR, 3.4–4.6). At the time of randomisation, women had been on ART for a median duration of 18 weeks (IQR, 12–23), 76% (*n =* 355) were suppressed to VL < 50 copies/ml, and 6% of women (*n =* 29) had VL ≥ 1,000 copies/ml. By design, 100% of women were breastfeeding at randomisation, and 91% (*n =* 430) reported EBF.

**Table 1:** Baseline characteristics of the study sample.

Characteristic	ART initiation under Option B+ (*n =* 381)		ART initiation under Option A and Option B+ (all participants) (*n =* 471)		Total (*n =* 471)
	Intervention: MCH-ART service (*n =* 186)	Control: standard of care (*n =* 195)	Intervention: MCH-ART service (*n =* 233)	Control: standard of care (*n =* 238)	
***At first antenatal clinic visit***					
Mean age (SD), years	28.9 (5.3)	28.3 (5.7)	28.6 (5.3)	28.5 (5.6)	28.6 (5.4)
Home language: isiXhosa	178 (96%)	191 (98%)	223 (96%)	233 (98%)	456 (97%)
Nulliparous	34 (18%)	35 (18%)	42 (18%)	45 (19%)	87 (18%)
Median gestational age at first ANC visit [IQR]	21 [16, 27]	21 [16, 27]	21 [16, 26]	21 [16, 27]	21 [16, 27]
Timing of first ANC visit					
First trimester	33 (18%)	34 (17%)	40 (17%)	40 (17%)	80 (17%)
Second trimester	107 (58%)	114 (58%)	142 (61%)	140 (59%)	282 (60%)
Third trimester	46 (25%)	47 (24%)	51 (22%)	58 (24%)	109 (23%)
Completed secondary/any tertiary education	43 (23%)	51 (26%)	53 (23%)	64 (27%)	117 (25%)
Currently employed	74 (40%)	73 (37%)	96 (41%)	88 (37%)	184 (39%)
Married/cohabiting	80 (43%)	70 (36%)	99 (42%)	94 (40%)	193 (41%)
Newly diagnosed HIV+ in this pregnancy	100 (54%)	117 (60%)	127 (55%)	141 (59%)	268 (57%)
Previous antiretrovirals: any	52 (28%)	47 (24%)	67 (29%)	61 (26%)	128 (27%)
Previous ART	8 (4%)	6 (3%)	11 (5%)	8 (3%)	19 (4%)
Previous zidovudine only	47 (25%)	42 (22%)	59 (25%)	54 (23%)	113 (24%)
Previous nevirapine	1 (0.5%)	2 (1%)	2 (0.9%)	4 (2%)	6 (1%)
Previous tuberculosis diagnosis	17 (9%)	16 (8%)	26 (11%)	26 (11%)	52 (11%)
Median CD4 count [IQR]; *n =* 459	348 [238, 507]	411 [279, 571]	327 [228, 486]	380 [260, 540]	354 [248, 517]
CD4 count					
≤200 cells/μl	30 (17%)	24 (13%)	40 (18%)	34 (15%)	74 (16%)
201–350 cells/μl	61 (34%)	51 (27%)	82 (36%)	69 (30%)	151 (33%)
>350 cells/μl	89 (49%)	114 (60%)	105 (46%)	129 (56%)	234 (51%)
Median log_10_ HIV VL [IQR]	4.0 [3.5, 4.6]	3.9 [3.1, 4.3]	4.0 [3.5, 4.6]	3.9 [3.2, 4.5]	4.0 [3.4, 4.6]
Log_10_ HIV VL					
<1,000 copies/ml	26 (14%)	41 (21%)	30 (13%)	47 (20%)	77 (16%)
1,000 to <10,000 copies/ml	69 (37%)	72 (37%)	81 (35%)	83 (35%)	164 (35%)
10,000 to <100,000 copies/ml	73 (39%)	64 (33%)	98 (42%)	84 (35%)	182 (39%)
≥100,000 copies/ml	18 (10%)	18 (9%)	24 (10%)	24 (10%)	48 (10%)
***At trial enrolment and randomisation***					
Median time on ART [IQR], weeks	18.1 [11.6, 22.7]	18.0 [12.1, 23.3]	18.6 [11.9, 22.6]	17.7 [12.1, 23.0]	18.0 [12.0, 22.9]
Initiated ART on day of first antenatal clinic visit	151 (81%)	170 (87%)	161 (69%)	178 (75%)	339 (72%)
Median days postpartum [IQR]	5 [4, 7]	5 [4, 8]	5 [4, 7]	5 [4, 8]	5 [4, 8]
Delivery					
In primary care	78 (42%)	76 (39%)	91 (39%)	95 (40%)	186 (39%)
Hospital care	102 (55%)	112 (57%)	136 (58%)	135 (57%)	271 (58%)
Born out of facility	6 (3%)	7 (4%)	6 (3%)	8 (3%)	14 (3%)
Missed ART dose reported in previous 30 days	30 (16%)	29 (15%)	33 (14%)	31 (13%)	64 (14%)
VL at randomisation (*n =* 470)					
<50 copies/ml	135 (73%)	150 (77%)	171 (73%)	184 (78%)	355 (76%)
50 to <1,000 copies/ml	37 (20%)	35 (18%)	43 (18%)	43 (18%)	86 (18%)
≥1,000 copies/ml	14 (8%)	9 (5%)	19 (8%)	10 (4%)	29 (6%)
Breastfeeding infant at time of randomisation	186 (100%)	195 (100%)	233 (100%)	238 (100%)	471 (100%)
Exclusively breastfed infant up to randomisation	169 (91%)	177 (91%)	211 (91%)	219 (92%)	430 (91%)

Data given as number (percent) unless otherwise indicated.

ANC, antenatal care; VL, viral load.

### Referral out of the MCH service

**Fig 2:**
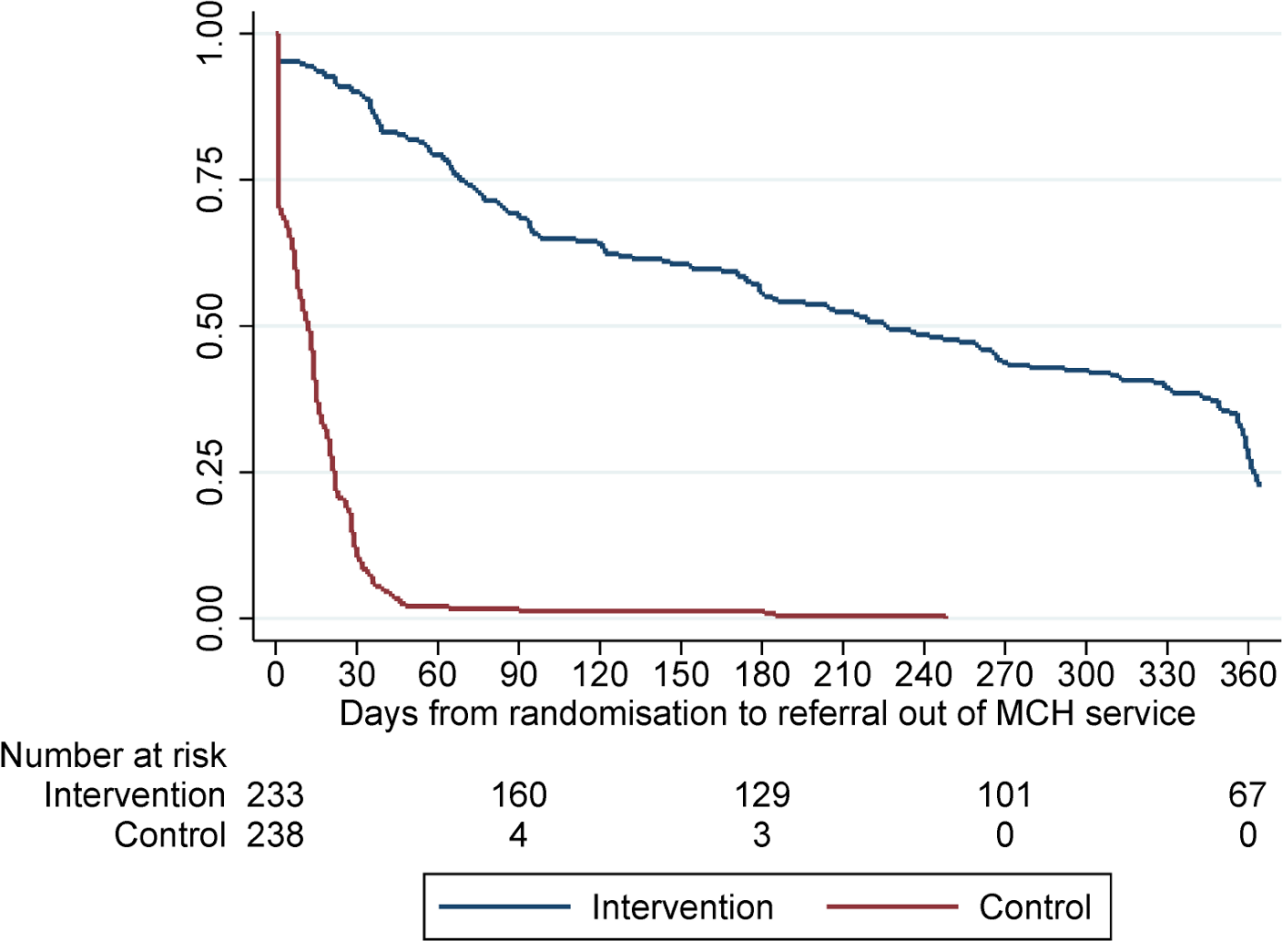
Time to referral out of the maternal and infant health (MCH) service for women in the intervention and control arms. Log-rank test, *p <* 0.001.

Fig 2 shows the distribution of time to referral out of the MCH service in the SOC versus MCH-ART arm. Among the 238 postpartum women (51%) allocated to be referred out of the MCH clinic immediately per SOC, the median time to referral out was 11 days (IQR, 1–21), and 90% of women were referred out within 30 days of delivery to 1 of 18 ART clinics. Women allocated to the intervention MCH-ART arm were retained in the MCH service until a median of 32 (IQR, 10–51) weeks postpartum.

### Follow-up in trial measurement visits

All women participating in the trial, regardless of trial arm, were followed with separate trial measurement visits up to 12 months postpartum through 21 January 2016. During this time, 59 women (13%) were lost from scheduled trial measurement visits (independent of their retention in routine HIV care), including 5 women who withdrew (1%) and 1 maternal death (<1%). Of the 412 women who completed the study outcome visit, 1 woman was missing VL measurement, and thus 411 women were available for the primary outcome (87% of those randomised; Fig 1). There was no difference in the completion of trial follow-up and availability of VL outcome data by trial arm or PMTCT option, but on average women who completed the 12-month trial measurement visit were older, were less likely to be diagnosed with HIV during their most recent pregnancy, and entered ANC at an earlier gestational age, compared to women lost from scheduled trial measurement visits (S1 Table). The median duration of ART use at the time of outcome assessment was 15.9 months (IQR, 14.5–17.3; median, 16.1 and 15.9 months in the intervention and control arms, respectively).

### Primary outcome

Table 2 compares the primary outcome overall and in its constituents between the trial arms, and shows almost identical results in the Option B+ population and the total trial population. Among the 411 women in the total trial population who completed trial follow-up and had VL data available, 77% of women (*n =* 155) randomised to the MCH-ART arm achieved the primary composite outcome of retention in ART services with VL < 50 copies/ml at 12 months postpartum, compared to 56% (*n =* 117) of women randomised to the SOC arm (risk difference, 0.21; 95% CI: 0.12–0.30; risk ratio, 1.37; 95% CI: 1.19–1.58; *p <* 0.001). Within this composite outcome, 80% of women (*n =* 330) had evidence of retention in ART services at 12 months postpartum: 88% (*n =* 178) in the MCH-ART arm and 72% (*n =* 151) in the SOC arm (*p <* 0.001). When the analytic population for the retention outcome was extended to all 471 women randomised (regardless of the availability of VL data), these proportions were 81% and 71%, respectively (*p =* 0.013). The observed differences between the MCH-ART and SOC arms persisted, for both retention in care and VS, under various definitions of these outcomes. For example, 80% of women (*n =* 162) in the MCH-ART arm and 68% (*n =* 142) in the SOC arm had VL < 1,000 copies/ml at the study outcome visit (*p =* 0.005), and the median times to VL ≥ 50 and VL ≥ 1,000 copies/ml, among those with VL < 50 copies/ml at randomisation, were significantly longer among women in the MCH-ART arm compared to women in the SOC arm (S1 and S2 Figs). Moreover, the findings related to retention did not differ appreciably by the data source or window period used to define retention (Tables 2 and S2).

**Table 2:** Comparison of primary outcome and variants between trial arms through 12 months postpartum.

Outcome	ART initiation under Option B+			ART initiation under Option A and Option B+ (all participants)			Total: All participants
	Intervention: MCH-ART service	Control: standard of care	*p*-Value	Intervention: MCH-ART service	Control: standard of care	*p*-Value	
**Composite endpoint: evidence of maternal retention in HIV care and VL < 50 copies/ml at 12 months postpartum (*n =* 411)**	126 (79%)	97 (57%)	<0.001	155 (77%)	117 (56%)	<0.001	272 (66%)
**Retention in care (in all participants enrolled, *n =* 471)**							
Evidence of engagement in HIV care at 12 months postpartum from any source	150 (81%)	136 (70%)	0.018	188 (81%)	168 (71%)	0.013	356 (76%)
Evidence of engagement in HIV care at 12 months postpartum by data source							
Clinic records	128 (69%)	93 (48%)	<0.001	160 (69%)	117 (49%)	<0.001	277 (59%)
Laboratory records	126 (68%)	119 (61%)	0.199	158 (68%)	148 (62%)	0.210	306 (65%)
Pharmacy refill	126 (68%)	104 (53%)	0.005	160 (69%)	130 (55%)	0.002	290 (62%)
**Retention in care (in all participants retained at outcome visit with VL data available, *n =* 411)**							
Evidence of engagement in HIV care at 12 months postpartum from any source	141 (88%)	122 (71%)	<0.001	178 (88%)	151 (72%)	<0.001	329 (80%)
Evidence of engagement in HIV care at 12 months postpartum by data source							
Clinic records	123 (77%)	86 (50%)	<0.001	155 (77%)	109 (52%)	<0.001	264 (64%)
Laboratory records	117 (73%)	106 (62%)	0.035	148 (73%)	132 (63%)	0.034	280 (68%)
Pharmacy refill	121 (76%)	97 (57%)	<0.001	155 (77%)	121 (58%)	<0.001	276 (67%)
**VL (in all participants retained at outcome visit with VL data available, *n =* 411)**							
<50 copies/ml at 12 months postpartum	126 (79%)	97 (57%)	<0.001	155 (77%)	117 (56%)	<0.001	272 (66%)
<1,000 copies/ml at 12 months postpartum	132 (83%)	118 (69%)	0.005	162 (80%)	142 (68%)	0.005	304 (74%)
**Loss of viral suppression among participants with VL < 50 copies/ml at randomisation (*n* = 312)**							
Cumulative percent with at least 1 VL ≥ 50 copies/ml through 12 months postpartum	23%	46%	0.011	25%	47%	0.003	37%
Cumulative percent with at least 1 VL ≥ 1,000 copies/ml through 12 months postpartum	17%	34%	0.006	17%	34%	0.002	26%
Two consecutive VL ≥ 50 copies/ml through 12 months postpartum	14 (12%)	36 (28%)	0.002	17 (11%)	47 (29%)	<0.001	64 (21%)
Two consecutive VL ≥ 1,000 copies/ml through 12 months postpartum	10 (8%)	20 (15%)	0.119	12 (8%)	29 (18%)	0.011	41 (13%)

Data given as number (percent) unless otherwise indicated.

VL, viral load.

**Fig 3:**
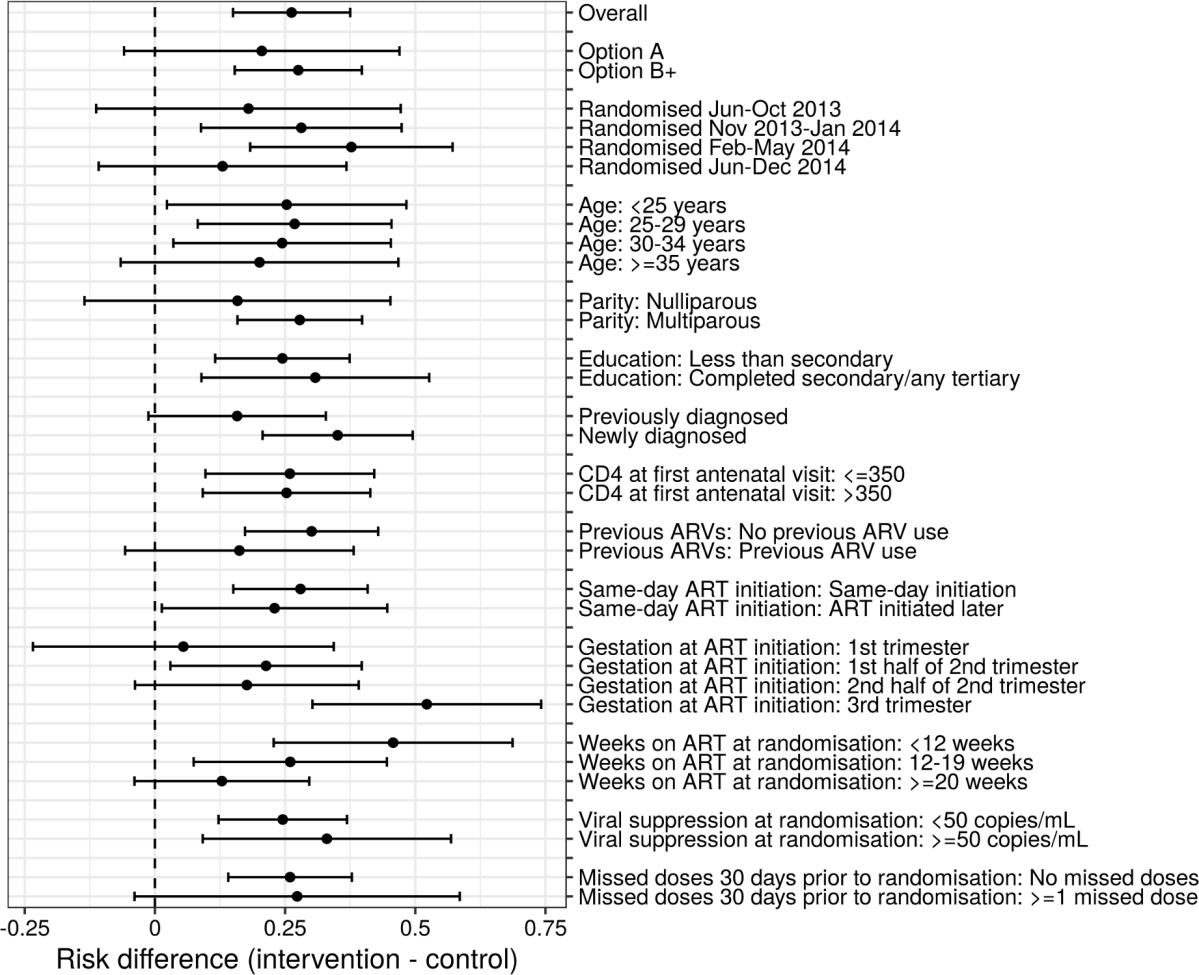
Forest plot of primary outcome across a priori subgroups of demographic and clinical characteristics. ARV, antiretroviral.

The overall findings for superior retention in care and virologic outcomes among women in the MCH-ART arm appeared consistent across subgroups of participants according to demographic and clinical characteristics (Figs 3, S3 and S4). Of note, the strongest intervention effects were observed in women initiating ART during the third trimester of pregnancy (risk difference, intervention minus control, 42%) compared to the second and first trimesters (risk differences, 16% and 4%, respectively). In a multivariable model (Table 3), the overall difference in the primary outcome between intervention and control persisted. In addition, ART initiation later in pregnancy, previous diagnosis with tuberculosis infection, and increased VL at the time of randomisation were associated with decreased VS and/or retention postpartum. In a secondary analysis with missing VL outcome data imputed, the observed differences between the intervention and control arms persisted (S3 Table).

**Table 3:** Results of additive binomial models examining the association between trial arm and primary outcome adjusted for demographic and clinical characteristics (*n =* 411).

Characteristic	Unadjusted models		Adjusted model	
	Risk difference	95% confidence interval	Risk difference	95% confidence interval
Trial arm (intervention minus control)	0.208	0.108 to 0.308	0.163	0.076 to 0.251
Maternal age (years)	0.016	0.007 to 0.023	0.013	0.009 to 0.017
Married or cohabiting (versus single)	0.102	−0.000 to 0.204	0.026	0.003 to 0.049
Newly diagnosed with HIV during pregnancy	0.033	−0.071 to 0.138	0.034	−0.021 to 0.089
Gestational age at ART initiation (weeks)	−0.007	−0.014 to −0.001	−0.010	−0.013 to −0.006
ART initiation under Option B+ (versus Option A)	0.061	−0.076 to 0.198	0.025	−0.089 to 0.140
Previous TB diagnosis (versus no previous TB diagnosis)	−0.183	−0.362 to −0.005	−0.149	−0.288 to −0.011
Viral load at randomisation (log_10_ copies/ml)	−0.178	−0.240 to −0.117	−0.157	−0.189 to −0.125
Duration of ART use at time of outcome assessment (weeks)	0.001	−0.004 to 0.007	−0.006	−0.010 to −0.002

Adjusted model includes all covariates shown.

TB, tuberculosis.

### Effect of duration of MCH-ART service

S4 Table compares outcomes among women randomised to the intervention who were retained in the MCH-ART service for different durations postpartum. Women randomised to the intervention who stopped breastfeeding and were referred out within 3 months postpartum had VL and ART retention outcomes measured at 12 months postpartum that were similar to those of women randomised to the control arm. Conversely, women who breastfed and were retained for longer periods in the MCH-ART service experienced improvements in outcomes measured at 12 months postpartum in a graded manner, with the largest intervention effects observed in women retained for at least 9 months postpartum; this trend persisted after adjusting for demographic and clinical covariates (S5 Table). Among women in the intervention arm, each additional month spent in the integrated MCH-ART service was associated with a 2.5% increase (95% CI: 1.9%–3.1%) in the absolute risk of being retained in care and virally suppressed at 12 months postpartum in adjusted models.

### Secondary outcomes

**Fig 4:**
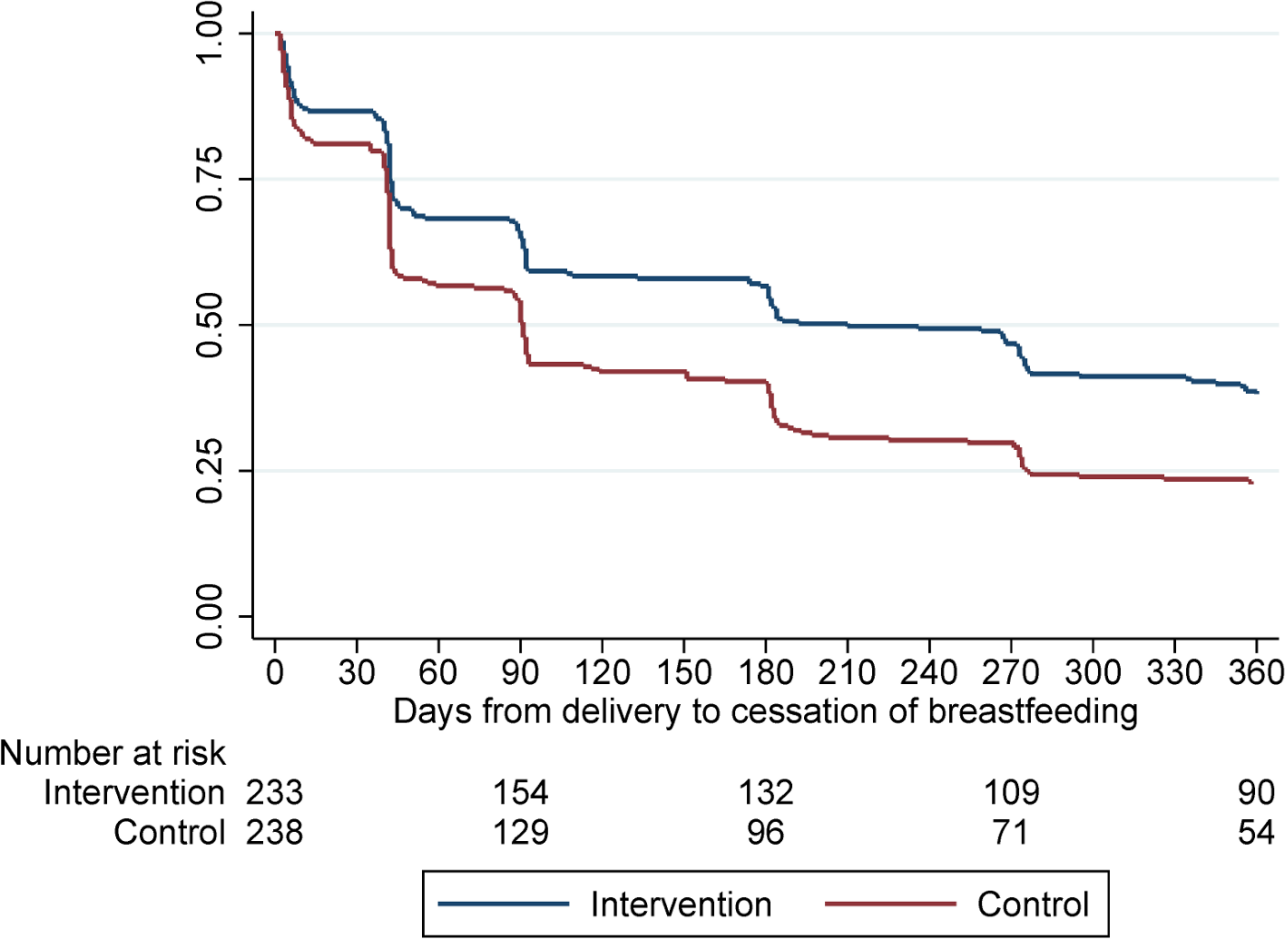
Time to cessation of any breastfeeding for women in the intervention and control arms. Log-rank test, *p =* 0.006.

**Fig 5:**
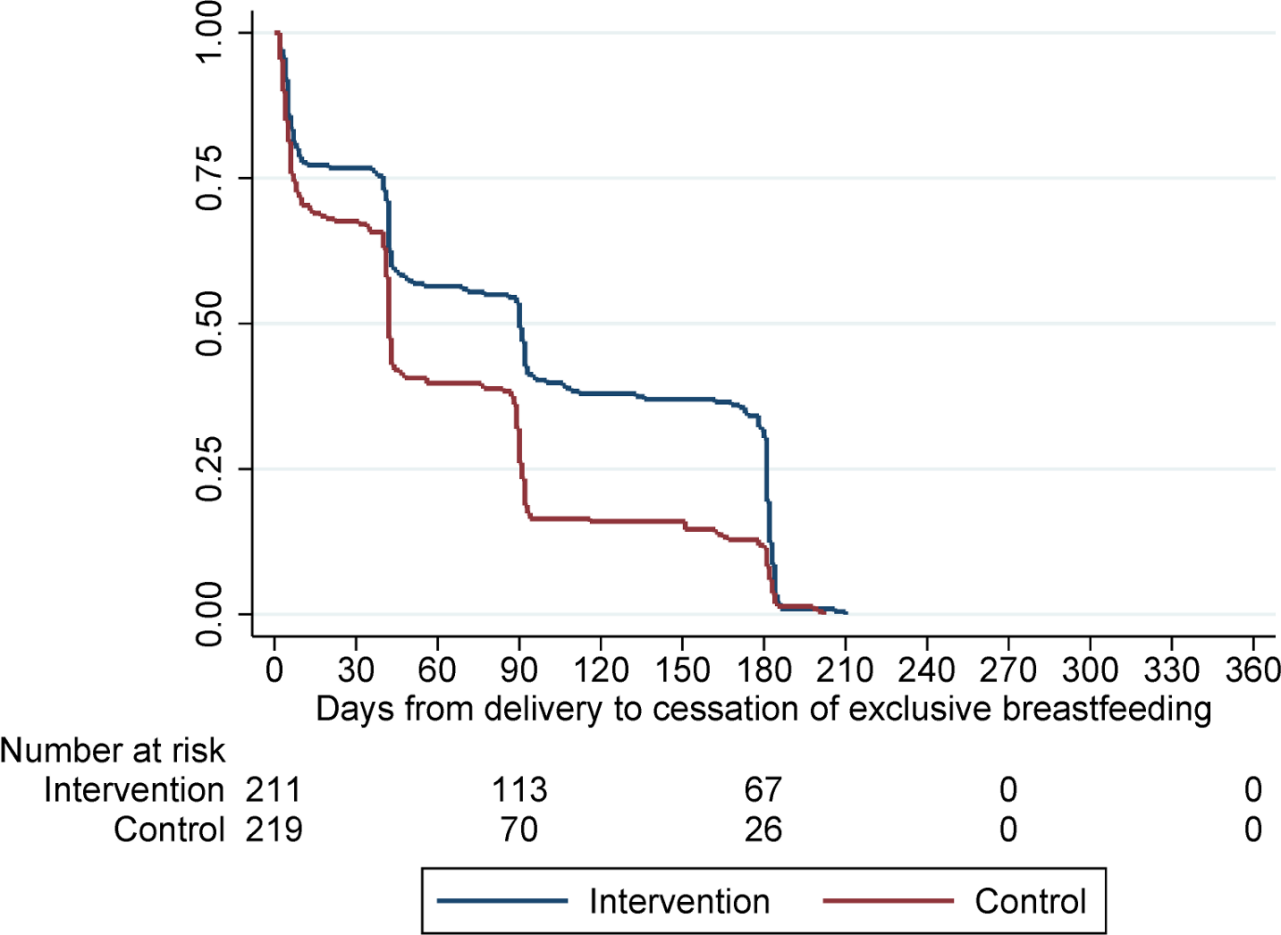
Time to cessation of exclusive breastfeeding for women in the intervention and control arms. Log-rank, *p <* 0.001.

Patterns of breastfeeding during the study differed between women randomised to the intervention and control arms (Figs 4 and 5). The median duration of any breastfeeding was longer among women randomised to the MCH-ART arm than among those randomised to the SOC arm in the total trial population (6.9 versus 3.0 months; *p =* 0.006) and when restricted to women initiating ART under Option B+ (6.0 versus 3.0 months; *p =* 0.023). By 12 months postpartum, 16% in the MCH-ART arm and 12% in the SOC arm reported still breastfeeding in the total trial population. The duration of EBF was shorter but followed a similar pattern, with a median of 3.0 months in the MCH-ART arm versus 1.4 months in the SOC arm (*p <* 0.001) in the total trial population.

Table 4 compares MCH outcomes between trial arms, which did not differ significantly between the intervention and control arms. Among 478 infants in the study, there were 9 deaths and 5 HIV infections observed in the first 12 months postpartum. Overall HIV-free survival through 12 months of age was 97%: mother-to-child HIV transmission at 12 months was 1.2% overall (comparison by trial arm, *p =* 0.178), and infant mortality was 1.9% (comparison by trial arm, *p =* 0.296).

**Table 4:** Comparison of secondary outcomes between the intervention and control arms.

Outcome	Total: all participants	ART initiation under Option B+			ART initiation under Option A and Option B+ (all participants)		
		Intervention: MCH-ART service	Control: standard of care	*p-*Value	Intervention: MCH-ART service	Control: standard of care	*p-*Value
Number of infants born to study	478	187	199		236	242	
Number of infant deaths	9^a^	5	3		6^a^	3^a^	
Infant mortality through 12 months postpartum	0.019	0.027	0.015	0.419	0.025	0.012	0.296
Number of infants with HIV diagnostic test results available	461	183	193		229	232	
Number of infant HIV infections	5^b^	3	1		4^b^	1^b^	
Infant HIV transmission through 12 months postpartum	0.012	0.018	0.007	0.304	0.019	0.005	0.178
Routine 6–10-week infant HIV diagnostic test results recorded in Road to Health Booklet^c^	189 (76%)	102 (88%)	51 (58%)	<0.001	127 (87%)	62 (60%)	<0.001
Co-trimoxazole received at 6 weeks of age (among breastfed infants)^d^	329 (83%)	144 (88%)	131 (82%)	0.217	174 (85%)	155 (80%)	0.206
Maternal family planning use at 12 months postpartum^e^	341 (83%)	138 (87%)	139 (81%)	0.107	169 (85%)	172 (82%)	0.483

Data given as number (percent) unless otherwise indicated.

^a^Infant deaths at 26, 30, 54, 62, 187, and 317 days of age (*n =* 6) in the intervention arm and at 45, 145, and 365 days (*n =* 3) in the control arm.

^b^Infants testing HIV-positive at 11, 12, 101, and 220 days of age (*n =* 4) in the intervention arm and at 308 days (*n =* 1) in the control arm.

^c^Of *n* = 249 infants with routine HIV diagnostic testing conducted in laboratory.

^d^Of *n* = 398 infants breastfed at 6 weeks postpartum and thus eligible for co-trimoxazole.

^e^Of *n* = 411 women with complete endpoint data.

## Discussion

This study demonstrates that integration of ART services into the MCH platform can lead to significant improvements in HIV+ women’s retention in care and VS up to 12 months postpartum. We also found longer durations of any BF and EBF among women who were randomised to integrated MCH and ART services. Overall, the integration of MCH and ART services may represent a simple intervention that can enhance the health of HIV+ women and their children in the first year after delivery.

This is one of the few randomised studies presenting evidence that integration of MCH and ART services can enhance women’s engagement in HIV care during the postnatal period and improve biomedical outcomes. While the challenges of retention in care and adherence to ART in HIV+ women during the postnatal period have been widely documented, rigorously evaluated interventions to strengthen women’s engagement in services are scant. Existing intervention modalities typically target individual women as the locus of intervention, for example through intensified patient counselling, mobile health interventions, services offered by lay health workers, and/or incentivising health behaviours, often with mixed results over short durations postpartum [11]. While these approaches may be useful to address single, specific causes of women’s disengagement from care, the intervention effects observed to date have been modest, and the feasibility of scaling up these interventions remains unclear.

In contrast, the integration of services represents a structural health systems intervention that may impact on multiple aspects of service engagement. We observed strong associations involving the primary outcome, women’s retention in care, and VS, as well as infant feeding practices; we had limited power to detect associations with outcomes that occur more commonly (such as maternal family planning use) or rarely (such as mother-to-child HIV transmission) in this setting. While not all research has shown improvements in care associated with integration of HIV and MCH services [22], our results are congruent with previous findings that models of care for postnatal ART services can influence outcomes for HIV+ women and their families. One health services evaluation from Malawi suggested that clinics that combined maternal and infant care yielded superior maternal retention outcomes compared to disaggregated forms of care where mothers received treatment within general adult ART services [14]. Similarly, a randomised controlled trial in Nigeria found significant increases in maternal retention in HIV care up to 3 months postpartum with a multicomponent package intervention that included co-scheduling of care for HIV+ mothers and their infants [23]. Building on these results, our trial findings advance the state of knowledge by providing compelling evidence demonstrating the specific effects of integrated postnatal services on multiple MCH outcomes.

The differences observed in the primary outcome between the intervention and control arms were generally similar across a range of subgroups. Of note, there is considerable interest in the impact of ART initiation on the same day as the first ANC visit, compared to delaying ART initiation to allow time for patient preparation [24]; we found the intervention effect persisted regardless of this timing. Moreover, it is plausible that women who are newly diagnosed with HIV in pregnancy may face particular challenges in ART retention and adherence, though intervention effects were similar regardless of when women were diagnosed with HIV infection. In addition, there are special concerns around the provision of lifelong ART for young pregnant and postpartum women [25], but here the intervention effects were consistent across all age groups.

We found that the strength of the intervention effect varied according to the amount of time postpartum spent in the intervention service in a graded manner: the largest improvements in the primary outcome were observed among women retained in the intervention beyond 9 months postpartum, with smaller but appreciable differences observed in women retained for 3 to 9 months. Meanwhile, among women in the intervention arm referred out to general ART services before 3 months postpartum, outcomes measured at 12 months postpartum were identical to those observed in the control arm. From these findings, we hypothesize that the integrated care intervention may provide a critical foundation for establishing and maintaining positive ART-related behaviours [26]. Related to this, we observed the strongest intervention effects in women who initiated ART later in pregnancy—a high-risk group that warrants special attention [27]. Specifically, women who started ART earlier in pregnancy, and thus spent more time establishing positive ART-related behaviours before delivery, may have benefitted less from remaining engaged in the integrated MCH-ART intervention service for longer durations postpartum. In contrast, women initiating ART later in pregnancy may have experienced greater benefit from the longer durations spent in the integrated service afforded by the intervention.

Alternate explanations for several findings warrant consideration. It is possible that some part of the intervention effect may be attributable to delaying the referral of women out of the MCH setting to adult ART clinics. Women retained in the intervention service after 9 months would have had less opportunity to disengage from care before the outcome visit at 12 months postpartum, though this would not explain the intervention effect observed among women referred out before 9 months. Here, we used 12 months postpartum for the primary outcome as most breastfeeding ends before this time in this setting, but it is critical to note that breastfeeding and mother-to-child transmission risk may be for longer periods, and challenges in maternal non-adherence and non-retention in services extend beyond this window. The transfer of patients between chronic care services is recognised as a high-risk window for non-adherence and non-retention [15,28], and longer durations of follow-up will be required to evaluate whether the effects of the intervention appear in keeping with this hypothesis. In addition, it is possible that differential availability of routine care data across health facilities in this setting could have explained some of the differences observed in retention outcomes between the intervention and control arms. However, we note consistency in the retention findings according to routine data source used, and further consistency with directly measured VS outcomes, making this explanation unlikely.

There are a range of mechanisms that may have contributed to the improved maternal health outcomes associated with the integrated services assessed here. The co-location of maternal and infant care may lower the burden of clinic visits, reducing the time and economic costs of healthcare attendance. For some women, receiving ART in the MCH setting may be associated with less HIV-related stigma compared to general adult ART services, and thus be more acceptable. In addition, nurse-midwives and other MCH providers may maintain better relationships with postpartum women, and in turn be better able to promote positive health behaviours. While these and other mechanisms warrant investigation, we posit that the reasons why integrated services lead to improved health outcomes are likely to be diverse and multifactorial, with different women benefitting from different aspects of integrated care for different reasons.

This study also provides novel evidence that integrated postnatal services are associated with prolonged breastfeeding, including EBF, by HIV+ women. Our finding may be unsurprising given the emphasis on breastfeeding within MCH services, in marked contrast to general adult services, where infant feeding practices may receive less attention [29]. While the relatively short overall duration of breastfeeding in this setting is in keeping with local norms, the trial’s findings of longer breastfeeding and higher levels of VS in women randomised to the MCH-ART intervention suggest that such integrated models of postnatal care may play an important role in implementing new WHO infant breastfeeding policies [21]. Furthermore, we found that the integrated MCH-ART service was associated with higher levels of recording of routine early infant diagnostic testing for HIV; given the challenges in implementing effective infant testing services in many settings [30], this is an important feature that warrants further investigation.

Several strengths and limitations require attention in interpreting these data. The trial took place in primary care public sector services in a single part of South Africa, and as with any health systems intervention, the generalisability of results should be considered with caution. The control condition used here needs particular consideration when seeking to extrapolate these results regarding the effects of integrated services. The SOC here may be broadly representative of how MCH and ART are provided across urban and high-burden areas of South Africa and southern Africa. However, this is not the SOC in all countries, and in different settings there may be other models of delivering integrated postnatal MCH and HIV care to mothers and infants, particularly in smaller primary care facilities. In this light, we suggest that the key findings here are for the value of integrated service delivery approaches for providing care in the postnatal period, rather than suggesting a single, universally ideal model of care. To evaluate the intervention, we used a novel composite primary endpoint incorporating both healthcare utilisation and the key biomarker of interest in HIV treatment, VL, though few interventions in this field have utilised such robust measures. We examined outcomes through 12 months postpartum—among the longest postpartum evaluations to date—but given that ART is lifelong, investigations of extended outcomes for both mothers and children are warranted. Here breastfeeding data were self-reported by mothers, raising ubiquitous concerns around biased reporting [31], though our study visits took place separately from routine care to help minimise such errors. Of note, this intervention does not address the phenomenon of loss to follow-up during pregnancy immediately after ART initiation, which is a separate and important consideration [8,14]. As discussed above, this trial only examines outcomes through 12 months postpartum, and the outcomes of transfers after that period require further investigation. Finally, the acceptability of service integration among patients and providers, as well as costs and cost-effectiveness, are important considerations not addressed in these data, but will be important avenues for future research.

In summary, these findings have substantial implications for policy and programme design. With few previous insights into the integration of HIV care into postnatal MCH settings, this study demonstrates that integrating ART services into the MCH platform for the duration of breastfeeding may be a simple and effective intervention for improving MCH outcomes in the context of HIV.

## Supporting information

S1 Fig Time to HIV viral load (VL) ≥ 50 copies/ml, among participants with VL < 50 copies/ml at randomisation. Log-rank test, *p =* 0.003.
(TIF)

S2 Fig Time to HIV viral load (VL) ≥ 1,000 copies/ml, among participants with VL < 50 copies/ml at randomisation. Log-rank test, *p =* 0.002.
(TIF)

S3 Fig Forest plot of primary outcome across a priori subgroups of demographic and clinical characteristics in the total study population. (TIF)

S4 Fig Forest plot of primary outcome across a priori subgroups of demographic and clinical characteristics among women initiating ART under Option B+. (TIF)

S1 Table Characteristics of participants completing study visits in person through 12 months, versus those not completing study visits through 12 months for any reason. (DOCX)

S2 Table Sensitivity analysis of retention in care outcomes re-analysed using retention outcome defined using windows of >9 to <15 months and >12 to <18 months postpartum. (DOCX)

S3 Table Results of multiple imputation of viral load data with adjusted results from additive binomial models (*n =* 471). Model includes all covariates shown.
(DOCX)

S4 Table Primary outcome measured at 12 months postpartum, among women randomised to the intervention arm, stratified by duration of retention in the integrated maternal and child health service. (DOCX)

S5 Table Results of additive binomial models examining the association between duration of time spent in the intervention arm and primary outcome adjusted for demographic and clinical risk factors (*n =* 411). (DOCX)

S6 Table CONSORT checklist. (DOC)

S1 Text Study protocol. (PDF)

## Abbreviations

ANC: antenatal care
ART: antiretroviral therapy
ARV: antiretrovira
EBF: exclusive breastfeeding
MCH: maternal and child health
NHLS: National Health Laboratory Services
PMTCT: prevention of mother-to-child HIV transmission
SOC: standard of care
VL: viral load
VS: viral suppression
