# Supplementary figures and images for: Integration of postpartum healthcare services for HIV-infected women and their infants in South Africa: A randomised controlled trial
Source: PLoS Med. 2018 Mar 30;15(3):e1002547. doi: 10.1371/journal.pmed.1002547 (PMC5877834; doi:10.1371/journal.pmed.1002547)

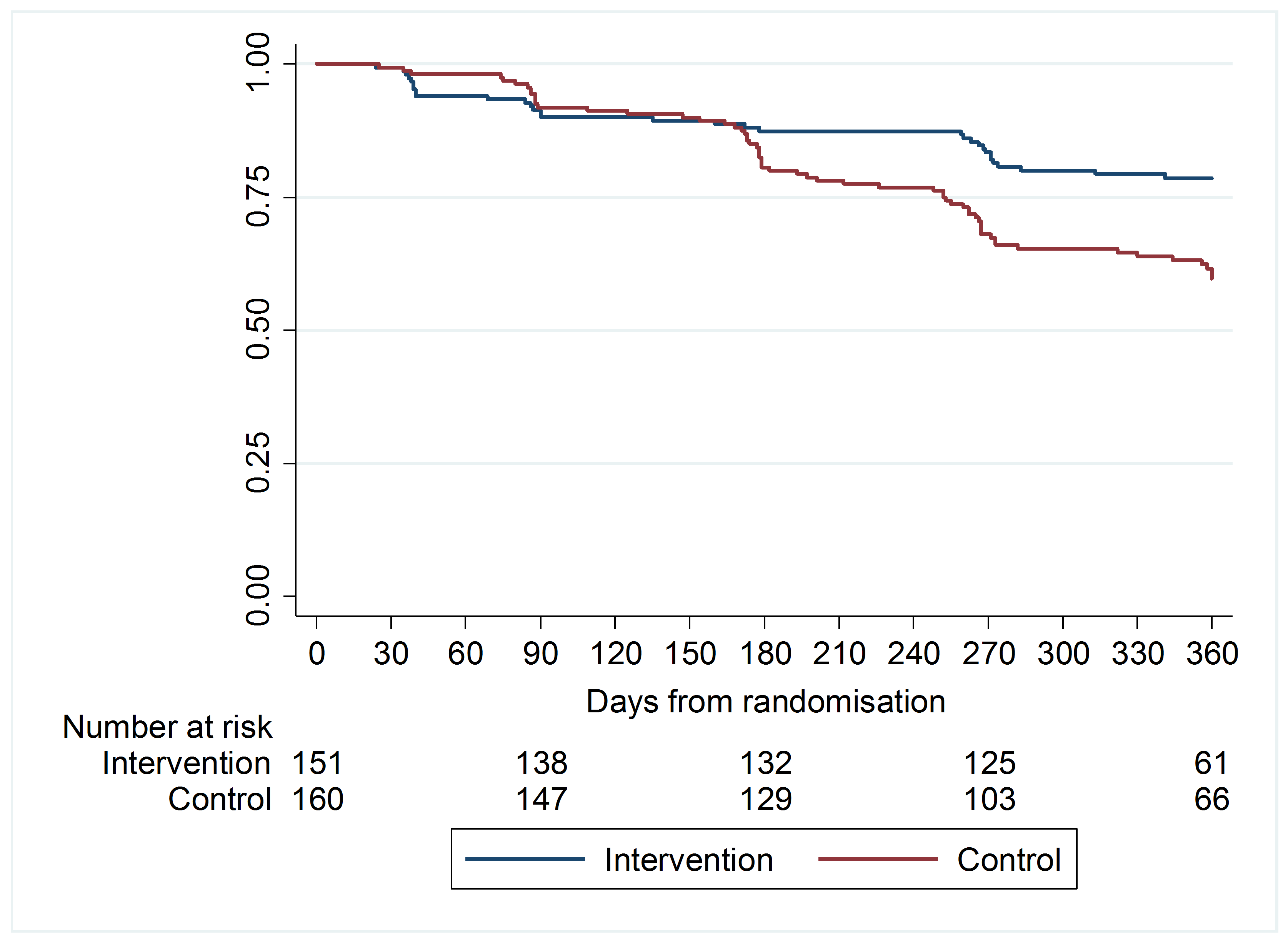

Supplement: S1 Fig — Log-rank test, p = 0.003. (TIF) [file pmed.1002547.s001.tif]

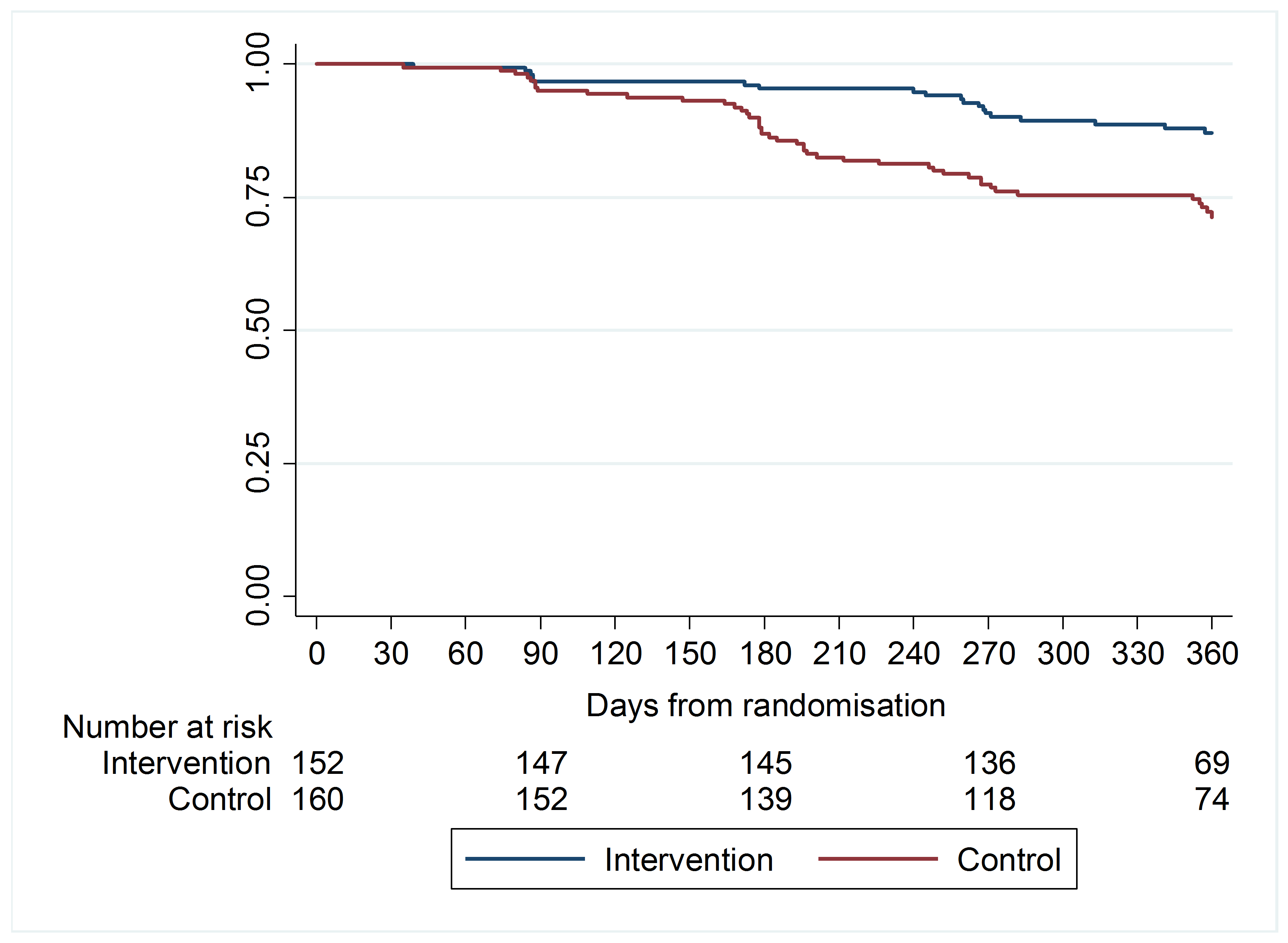

Supplement: S2 Fig — Log-rank test, p = 0.002. (TIF) [file pmed.1002547.s002.tif]

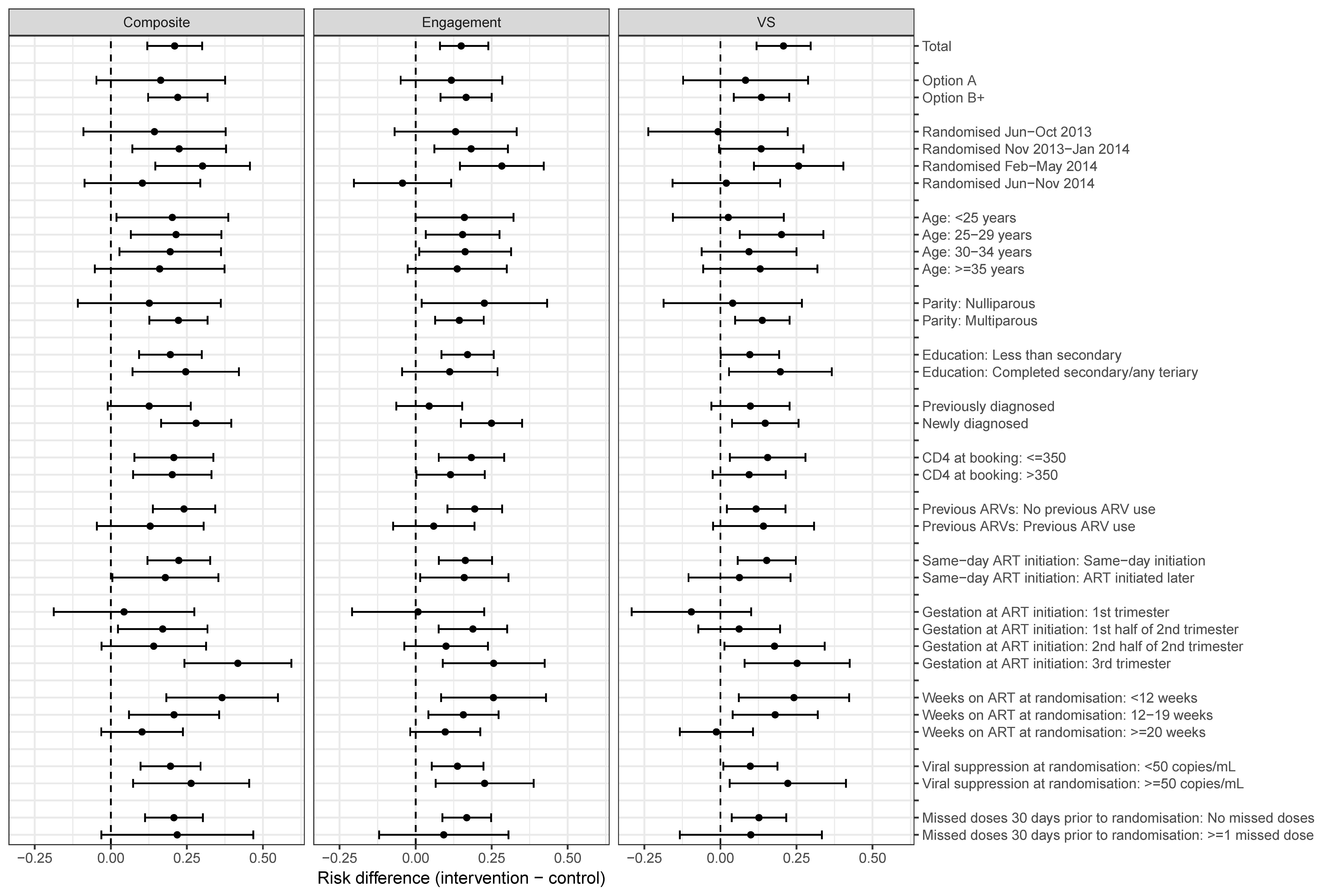

Supplement: S3 Fig — (TIF) [file pmed.1002547.s003.tif]

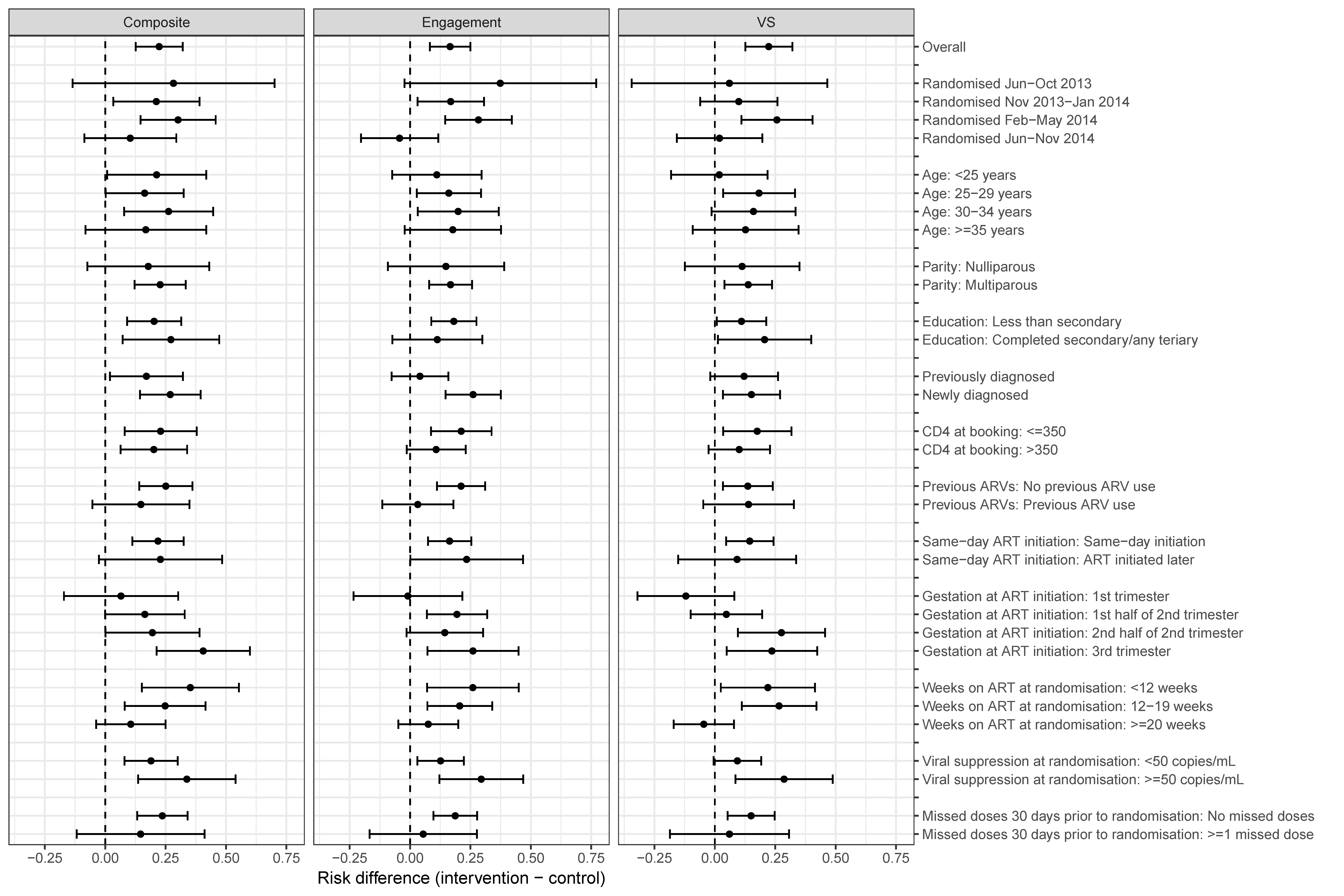

Supplement: S4 Fig — (TIF) [file pmed.1002547.s004.tif]
