# Supplementary material for: Integration of postpartum healthcare services for HIV-infected women and their infants in South Africa: A randomised controlled trial
Source: PLoS Med. 2018 Mar 30;15(3):e1002547. doi: 10.1371/journal.pmed.1002547 (PMC5877834; doi:10.1371/journal.pmed.1002547)
Supplement: S1 Table — (DOCX) [file pmed.1002547.s005.docx]

**S1 Table.** Characteristics of participants completing study visits in person through 12 months, versus those not completing study visits through 12 months for any reason.

|  | **ART initiation under Option B+** | | | **ART initiation under Option A and Option B+ (all participants) (n=471)** | | |
| --- | --- | --- | --- | --- | --- | --- |
|  | Completed study visit at/after 12m (n=332) | Lost to study before 12m (n=49) | p-value | Completed study visit at/after 12m (n=412) | Lost to study before 12m (n=59) | p-value |
| **Study arm** |  |  |  |  |  |  |
| Intervention | 160 (48) | 26 (53) |  | 202 (49) | 31 (53) |  |
| Control | 172 (52) | 23 (47) | 0.544 | 210 (51) | 28 (47) | 0.677 |
| **ART eligibility at ART initiation:** Option A |  |  |  | 80 (19) | 10 (17) |  |
| Option B+ | - | - | - | 332 (81) | 49 (83) | 0.726 |
| ***At first antenatal clinic visit*** |  |  |  |  |  |  |
| Mean age (SD) | 28.8 (5.5) | 27.1 (5.1) | 0.036 | 28.8 (5.5) | 26.8 (4.9) | 0.008 |
| Nulliparous | 52 (16) | 17 (35) | 0.003 | 66 (16) | 21 (36) | 0.001 |
| Median gestation at first ANC visit (IQR) | 21 [16, 26] | 23 [18, 30] | 0.150 | 21 [16, 26] | 24 [18, 30] | 0.049 |
| First trimester | 59 (18) | 8 (16) |  | 72 (17) | 8 (14) |  |
| Second trimester | 197 (59) | 24 (49) |  | 251 (61) | 31 (53) |  |
| Third trimester | 76 (23) | 17 (35) | 0.207 | 89 (22) | 20 (34) | 0.123 |
| Completed secondary education | 80 (24) | 14 (29) | 0.482 | 101 (25) | 16 (27) | 0.633 |
| Currently employed | 122 (37) | 25 (51) | 0.061 | 155 (38) | 29 (49) | 0.116 |
| Married/cohabiting | 133 (40) | 17 (35) | 0.533 | 170 (41) | 23 (39) | 0.779 |
| Newly diagnosed HIV+ in this pregnancy | 181 (55) | 36 (73) | 0.013 | 223 (54) | 45 (76) | 0.001 |
| Previous ARVs: Any | 89 (27) | 10 (20) | 0.387 | 117 (28) | 11 (19) | 0.121 |
| Previous ART | 13 (4) | 1 (2) | 1.000 | 18 (4) | 1 (2) | 0.491 |
| Previous AZT only | 80 (24) | 9 (18) | 0.470 | 103 (25) | 10 (17) | 0.196 |
| Previous NVP | 3 (0.9) | 0 (0) | 1.000 | 6 (1) | 0 (0) | 1.000 |
| Previous TB diagnosis | 30 (9) | 3 (6) | 0.784 | 48 (12) | 4 (7) | 0.374 |
| Median CD4 (IQR); *n*=459 | 372 [262, 544] | 418 [270, 531] | 0.440 | 346 [248, 507] | 418 [251, 546] | 0.124 |
| CD4 <=200 cells/µL | 48 (15) | 6 (12) |  | 68 (17) | 6 (10) |  |
| CD4 201-350 cells/µL | 100 (31) | 12 (24) |  | 135 (34) | 16 (27) |  |
| CD4 >350 cells/µL | 172 (54) | 31 (63) | 0.513 | 197 (49) | 37 (63) | 0.152 |
| Median log_10_ HIV VL (IQR) | 3.9 [3.3, 4.5] | 3.9 [3.1, 4.4] | 0.323 | 4.0 [3.4, 4.6] | 3.9 [3.0, 4.4] | 0.168 |
| VL<1000 copies/mL | 55 (17) | 12 (24) |  | 63 (15) | 14 (24) |  |
| VL 1000 - <10 000 copies/mL | 124 (37) | 17 (35) |  | 145 (35) | 19 (32) |  |
| VL 10 000 - <100 000 copies/mL | 121 (36) | 16 (33) |  | 161 (39) | 21 (36) |  |
| VL >=100 000 copies/mL | 32 (10) | 4 (8) | 0.624 | 43 (10) | 5 (8) | 0.451 |
| ***At trial enrolment and randomisation*** |  |  |  |  |  |  |
| Median time on ART (weeks) | 18.6 [12.2, 23.0] | 16.1 [11.1, 22.7] | 0.221 | 18.4 [12.3, 22.9] | 15.9 [10.1, 22.6] | 0.081 |
| Initiated ART on day of first antenatal clinic visit | 278 (84) | 43 (88) | 0.674 | 293 (71) | 46 (78) | 0.352 |
| Median days postpartum (IQR) | 5 [4, 7] | 5 [4, 7] | 0.755 | 5 [4, 8] | 5 [4, 8] | 0.642 |
| Delivered in primary care | 133 (40) | 21 (43) |  | 160 (39) | 26 (44) |  |
| Hospital care | 187 (56) | 27 (55) |  | 239 (58) | 32 (54) |  |
| Born out of facility | 12 (4) | 1 (2) | 0.960 | 13 (3) | 1 (2) | 0.713 |
| Missed ART dose reported in previous 30d | 52 (16) | 7 (14) | 1.000 | 57 (14) | 7 (12) | 0.840 |
| VL at randomisation (*n*=470) |  |  |  |  |  |  |
| <50 copies/mL | 248 (75) | 37 (76) |  | 312 (76) | 43 (73) |  |
| 50-1000 copies/mL | 65 (20) | 7 (14) |  | 76 (18) | 10 (17) |  |
| >1000 copies/mL | 18 (5) | 5 (10) | 0.272 | 23 (6) | 6 (10) | 0.381 |
| Exclusively breastfed infant up to randomisation | 304 (92) | 42 (86) | 0.188 | 379 (92) | 51 (86) | 0.212 |
