# Supplementary material for: Integration of postpartum healthcare services for HIV-infected women and their infants in South Africa: A randomised controlled trial
Source: PLoS Med. 2018 Mar 30;15(3):e1002547. doi: 10.1371/journal.pmed.1002547 (PMC5877834; doi:10.1371/journal.pmed.1002547)
Supplement: S2 Table — (DOCX) [file pmed.1002547.s006.docx]

**S2 Table.** Sensitivity analysis of retention in care outcomes re-analysed using retention outcome defined using windows of (i) >9 to <15 months and (ii) >12 to <18 months postpartum.

|  | **ART initiation under Option B+** | | | **ART initiation under Option A and Option B+ (all participants)** | | | **Total** |
| --- | --- | --- | --- | --- | --- | --- | --- |
|  | Intervention  MCH-ART service | Control  Standard of Care | p-value | Intervention  MCH-ART service | Control  Standard of Care | p-value | All participants |
| **Composite endpoint** |  |  |  |  |  |  |  |
| **Evidence of maternal retention in HIV care at 9-15 months *AND* viral load (VL) <50 copies/mL at 12 months postpartum (n=411)** | 116(73) | 86(50) | <0.001 | 139(69) | 104(50) | <0.001 | 243(59) |
| **Evidence of maternal retention in HIV care at 12-18 months *AND* viral load (VL) <50 copies/mL at 12 months postpartum (n=411)** | 101(63) | 79(46) | 0.002 | 125(62) | 98(47) | 0.002 | 223(54) |
| **Retention in care**  **(in all participants enrolled, n=471)** |  |  |  |  |  |  |  |
| Evidence of engagement in HIV care at 9-15 months postpartum from any source | 133(72) | 115(59) | 0.010 | 161(69) | 142 (60) | 0.033 | 303(64) |
| Evidence of engagement in HIV care at 12-18 months postpartum from any source | 123(66) | 105(54) | 0.014 | 150(64) | 133(56) | 0.060 | 283(60) |
|  |  |  |  |  |  |  |  |
| **Retention in care**  **(in all participants retained in trial measurement visits with VL data available, n=411)** |  |  |  |  |  |  |  |
| Evidence of engagement in HIV care at 9-15 months postpartum from any source | 128(80) | 106(62) | <0.001 | 156(77) | 131(63) | 0.001 | 287(70) |
| Evidence of engagement in HIV care at 12-18 months postpartum from any source | 116(73) | 97(57) | 0.003 | 143(71) | 123(59) | 0.011 | 266(65) |
|  |  |  |  |  |  |  |  |
