# Supplementary material for: Integration of postpartum healthcare services for HIV-infected women and their infants in South Africa: A randomised controlled trial
Source: PLoS Med. 2018 Mar 30;15(3):e1002547. doi: 10.1371/journal.pmed.1002547 (PMC5877834; doi:10.1371/journal.pmed.1002547)
Supplement: S3 Table — Model includes all covariates shown. (DOCX) [file pmed.1002547.s007.docx]

**S3 Table.** Results of multiple imputation of viral load data with adjusted results from additive binomial models (n=471). Model includes all covariates shown.

|  | *Risk difference* | *95% Confidence Interval* |
| --- | --- | --- |
| Trial arm: *Intervention minus control* | 0.177 | 0.090 to 0.265 |
| Maternal age (years) | 0.015 | 0.007 to 0.023 |
| Married or cohabiting (versus single) | 0.046 | -0.045 to 0.137 |
| Newly diagnosed with HIV during pregnancy | 0.005 | -0.086 to 0.096 |
| Gestation at ART initiation (weeks) | -0.009 | -0.017 to -0.002 |
| ART initiation under Option B+ (versus Option A) | 0.024 | -0.088 to 0.137 |
| Previous TB diagnosis (vs no previous TB diagnosis) | -0.154 | -0.296 to -0.013 |
| Viral load at randomisation (log10 copies/mL) | -0.159 | -0.234 to -0.084 |
| Duration of ART use at time of outcome assessment (weeks) | -0.005 | -0.012 to 0.001 |
