# Supplementary material for: Integration of postpartum healthcare services for HIV-infected women and their infants in South Africa: A randomised controlled trial
Source: PLoS Med. 2018 Mar 30;15(3):e1002547. doi: 10.1371/journal.pmed.1002547 (PMC5877834; doi:10.1371/journal.pmed.1002547)
Supplement: S4 Table — (DOCX) [file pmed.1002547.s008.docx]

**S4 Table.** Primary outcome measured at 12 months postpartum, among women randomised to the intervention arm, stratified by duration of retention in the integrated maternal & child health service.

|  | **Duration of retention in integrated MCH-ART service** | | | | **All women in intervention arm** | **All women in control arm** |
| --- | --- | --- | --- | --- | --- | --- |
|  | *<3 months* | *≥3 to <6 months* | *≥6 to <9 months* | *≥9 months* |  |  |
| Number of women (as randomised) | 74 | 31 | 27 | 101 | 233 | 238 |
| Number of women with VL outcome data available | 54 (73) | 28 (90) | 21 (78) | 99 (98) | 202 (87) | 209 (88) |
| Retained in care and VL<50 copies/mL at 12 months postpartum (among women with 12-month outcome data available) | 29 (54) | 19 (68) | 15 (71) | 92 (93) | 155 (77) | 117 (56) |
| Retained in care at 12 months postpartum (among women with 12-month outcome data available) | 39 (72) | 21 (75) | 19 (90) | 99 (100) | 178 (88) | 151 (72) |
| Retained in care at 12 months postpartum (among all women randomised) | 45 (61) | 21 (68) | 21 (78) | 101 (100) | 188 (81) | 168 (71) |
| VL<50 copies/mL (among women with 12-month outcome data available) | 29 (54) | 19 (68) | 15 (71) | 92 (93) | 155 (77) | 117 (56) |
