# Supplementary material for: Integration of postpartum healthcare services for HIV-infected women and their infants in South Africa: A randomised controlled trial
Source: PLoS Med. 2018 Mar 30;15(3):e1002547. doi: 10.1371/journal.pmed.1002547 (PMC5877834; doi:10.1371/journal.pmed.1002547)
Supplement: S5 Table — (DOCX) [file pmed.1002547.s009.docx]

**S5 Table.** Results of additive binomial models examining the association between duration of time spent in the intervention arm and primary outcome adjusted for demographic and clinical risk factors (n=411)

|  | *Unadjusted models* | | *Adjusted model ** | |
| --- | --- | --- | --- | --- |
|  | *Risk difference* | *95% Confidence Interval* | *Risk difference* | *95% Confidence Interval* |
| Reference category (control arm): *Intervention minus control* | 1.0 |  | 1.0 |  |
| <3 months in intervention service | -0.023 | -0.201 to 0.155 | 0.001 | -0.142 to 0.144 |
| ≥3 to <6 months in intervention service | 0.119 | -0.097 to 0.335 | 0.108 | -0.115 to 0.332 |
| ≥6 to <9 months in intervention service | 0.154 | -0.081 to 0.390 | 0.162 | -0.122 to 0.446 |
| ≥9 months in intervention service | 0.370 | 0.282 to 0.457 | 0.238 | -0.138 to 0.614 |

* Adjusted for all covariates shown in supplementary table 2: maternal age, marital status, timing of HIV diagnosis, gestation at ART initiation, ART initiation under Option B+ (versus Option A), previous TB diagnosis, viral load at randomisation, and duration of ART use at time of outcome assessment.
