# Supplementary material for: Integration of postpartum healthcare services for HIV-infected women and their infants in South Africa: A randomised controlled trial
Source: PLoS Med. 2018 Mar 30;15(3):e1002547. doi: 10.1371/journal.pmed.1002547 (PMC5877834; doi:10.1371/journal.pmed.1002547)
Supplement: S6 Table — (DOC) [file pmed.1002547.s010.doc]

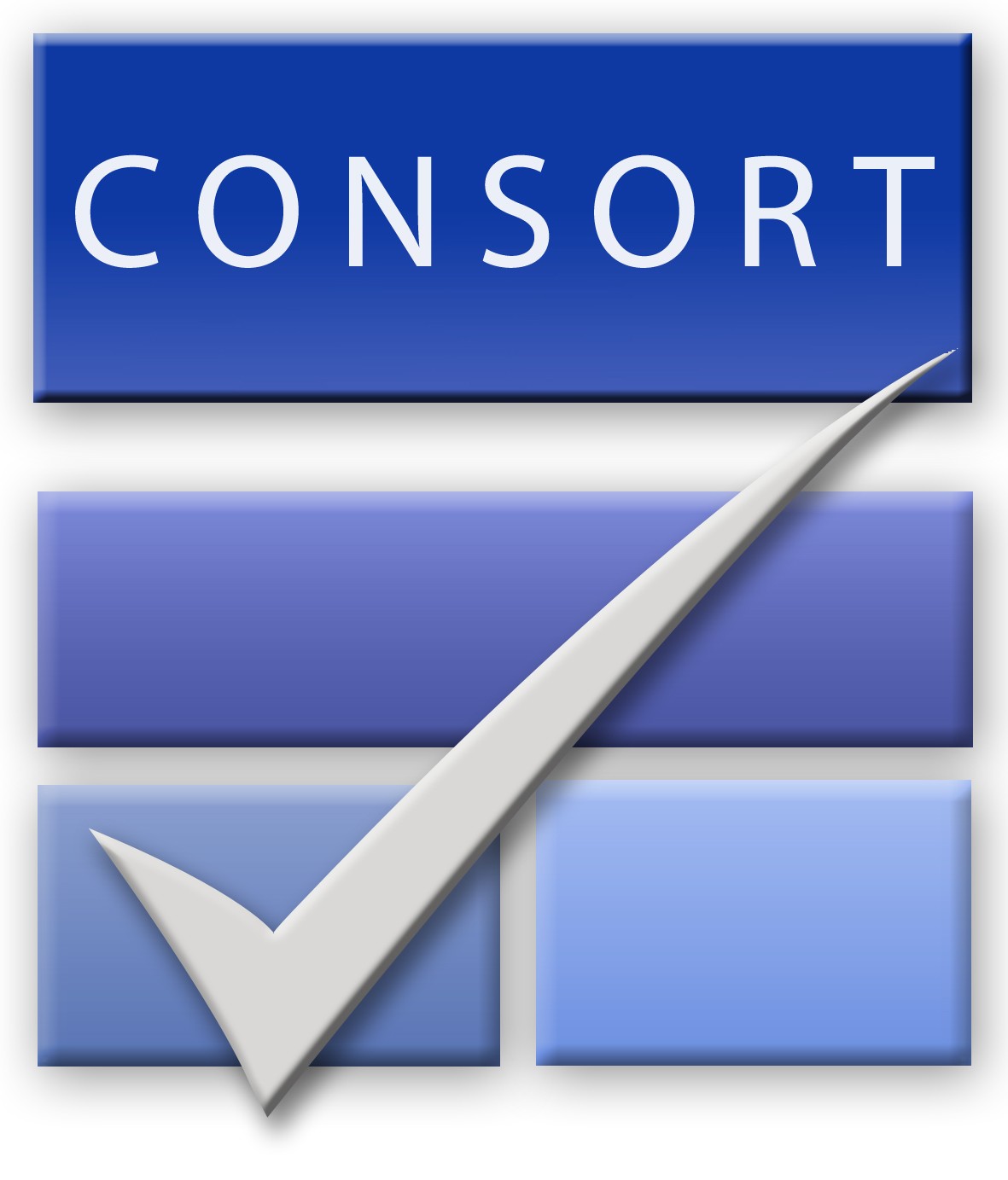
CONSORT 2010 checklist of information to include when reporting a randomised trial

**Integration of postpartum health care services for HIV-infected women and their infants in South Africa: a randomised controlled trial**

| Section/Topic | Item No | Checklist item | Reported on page No |
| --- | --- | --- | --- |
| Title and abstract | | | |
|  | 1a | Identification as a randomised trial in the title | Title page |
|  | 1b | Structured summary of trial design, methods, results, and conclusions (for specific guidance see CONSORT for abstracts) | As per journal guidance on page called “abstract” |
| Introduction | | | |
| Background and objectives | 2a | Scientific background and explanation of rationale | Introduction, paras 1-3 |
|  | 2b | Specific objectives or hypotheses | Introduction, para 3 |
| Methods | | | |
| Trial design | 3a | Description of trial design (such as parallel, factorial) including allocation ratio | Methods, para 1 and 4 |
|  | 3b | Important changes to methods after trial commencement (such as eligibility criteria), with reasons | Methods paras 2 and 14 |
| Participants | 4a | Eligibility criteria for participants 5 | Methods para 3 |
|  | 4b | Settings and locations where the data were collected | Methods para 1+2 |
| Interventions | 5 | The interventions for each group with sufficient details to allow replication, including how and when they were actually administered | Methods para 5-6-7 |
| Outcomes | 6a | Completely defined pre-specified primary and secondary outcome measures, including how and when they were assessed | Methods para 13 |
|  | 6b | Any changes to trial outcomes after the trial commenced, with reasons | n/a |
| Sample size | 7a | How sample size was determined | Methods para 14 |
|  | 7b | When applicable, explanation of any interim analyses and stopping guidelines | n/a |
| Randomisation: |  |  |  |
| Sequence generation | 8a | Method used to generate the random allocation sequence | Methods para 4 |
|  | 8b | Type of randomisation; details of any restriction (such as blocking and block size) | Methods para 4 |
| Allocation concealment mechanism | 9 | Mechanism used to implement the random allocation sequence (such as sequentially numbered containers), describing any steps taken to conceal the sequence until interventions were assigned | Methods para 4 |
| Implementation | 10 | Who generated the random allocation sequence, who enrolled participants, and who assigned participants to interventions | Methods para 4 |
| Blinding | 11a | If done, who was blinded after assignment to interventions (for example, participants, care providers, those assessing outcomes) and how | Methods para 10 |
|  | 11b | If relevant, description of the similarity of interventions | Methods paras 5-6-7 |
| Statistical methods | 12a | Statistical methods used to compare groups for primary and secondary outcomes | Methods para 15 |
|  | 12b | Methods for additional analyses, such as subgroup analyses and adjusted analyses | Methods para 15 |
| Results | | | |
| Participant flow (a diagram is strongly recommended) | 13a | For each group, the numbers of participants who were randomly assigned, received intended treatment, and were analysed for the primary outcome | Results para 1, Figure 1 |
|  | 13b | For each group, losses and exclusions after randomisation, together with reasons | Results para 4, Figure 1 |
| Recruitment | 14a | Dates defining the periods of recruitment and follow-up | Results para 1 |
|  | 14b | Why the trial ended or was stopped | Results para 1 |
| Baseline data | 15 | A table showing baseline demographic and clinical characteristics for each group | Table 1 |
| Numbers analysed | 16 | For each group, number of participants (denominator) included in each analysis and whether the analysis was by original assigned groups | Fig 1, Table 1, results para 5 |
| Outcomes and estimation | 17a | For each primary and secondary outcome, results for each group, and the estimated effect size and its precision (such as 95% confidence interval) | Results para 5, table 2 |
|  | 17b | For binary outcomes, presentation of both absolute and relative effect sizes is recommended | Results para 5-6-7-8 |
| Ancillary analyses | 18 | Results of any other analyses performed, including subgroup analyses and adjusted analyses, distinguishing pre-specified from exploratory | Results para 7; Figure 3 supplementary materials |
| Harms | 19 | All important harms or unintended effects in each group (for specific guidance see CONSORT for harms) | Results para 8-9 |
| Discussion | | | |
| Limitations | 20 | Trial limitations, addressing sources of potential bias, imprecision, and, if relevant, multiplicity of analyses | Discussion para 10 |
| Generalisability | 21 | Generalisability (external validity, applicability) of the trial findings | Discussion para 8 |
| Interpretation | 22 | Interpretation consistent with results, balancing benefits and harms, and considering other relevant evidence | Discussion para 2-3-4 |
| Other information | | |  |
| Registration | 23 | Registration number and name of trial registry | Methods para 11 |
| Protocol | 24 | Where the full trial protocol can be accessed, if available | Methods para 11 |
| Funding | 25 | Sources of funding and other support (such as supply of drugs), role of funders | Financial disclosure field per journal |

*We strongly recommend reading this statement in conjunction with the CONSORT 2010 Explanation and Elaboration for important clarifications on all the items. If relevant, we also recommend reading CONSORT extensions for cluster randomised trials, non-inferiority and equivalence trials, non-pharmacological treatments, herbal interventions, and pragmatic trials. Additional extensions are forthcoming: for those and for up to date references relevant to this checklist, see [www.consort-statement.org](http://www.consort-statement.org/).
