# Supplementary material for: Integration of postpartum healthcare services for HIV-infected women and their infants in South Africa: A randomised controlled trial
Source: PLoS Med. 2018 Mar 30;15(3):e1002547. doi: 10.1371/journal.pmed.1002547 (PMC5877834; doi:10.1371/journal.pmed.1002547)
Supplement: S1 Text — (PDF) [file pmed.1002547.s011.pdf]

Strategies to optimize antiretroviral therapy services for maternal & child health: the MCH-ART study

Principal Investigators

Prof Elaine Abrams
ICAP, Mailman School of Public Health
Columbia University

A/Prof Landon Myer
School of Public Health & Family Medicine
University of Cape Town

Co-Investigators

A/Prof Linda-Gail Bekker
Desmond Tutu HIV Centre, IIDMM
University of Cape Town

Ms Allison Zerbe
ICAP, Mailman School of Public Health
Columbia University

Dr Greg Petro
Metro-West Director: O&G
Provincial Government of the Western Cape

Dr David Pienaar
HAST Directorate
Provincial Government of the Western Cape

A/Prof Francesca Little
Department of Statistical Sciences
University of Cape Town

Prof Heather Zar
Department of Paediatrics & Child Health
University of Cape Town
Red Cross War Memorial Children's Hospital

Prof James McIntyre
Anova Health Institute & School of Public Health & Family Medicine, UCT

Prof Robert Remien
HIV Centre for Clinical & Behavioural Studies
New York State Psychiatric Institute
Columbia University

Prof Claude Mellins
HIV Centre for Clinical & Behavioural Studies
New York State Psychiatric Institute
Columbia University

Dr Catherine Orrell
Desmond Tutu HIV Centre, IIDMM
University of Cape Town

14 January 2015

Study synopsis

During the past decade there have been major advances in the provision of services for the prevention of mother-to-child transmission of HIV infection (PMTCT) in South Africa (SA). Still, there were more than 40 000 new paediatric HIV infections in SA during 2010, accounting for more than 10% of the global total. The effectiveness of PMTCT interventions is based on use of antiretroviral drugs to suppress maternal viraemia and/or for prophylaxis to the fetus/newborn for the duration of exposure to maternal HIV, including during gestation, labour and delivery, and throughout the period of breastfeeding.

Antiretroviral therapy (ART) is the central intervention to promote maternal and child health (MCH) in the context of HIV infection. Women with advanced HIV disease who are eligible for ART (based on CD4 cell counts <350 cells/mm3 and/or WHO staging) are at the greatest risk of both maternal morbidity and vertical HIV transmission. Use of ART during pregnancy with high levels of adherence, throughout gestation and postpartum, are required to achieve viral suppression in order to minimize MTCT risk and promote maternal health. In particular, the postpartum period is becoming increasingly important for PMTCT because of recent developments in national policy that now promote exclusive breastfeeding by HIV-infected women, on the assumption that they are virally suppressed. Yet there is growing evidence from across SA that the postpartum adherence to ART and retention in care of women who initiate ART during pregnancy is suboptimal, thus increasing the risk of MTCT.

In this light, there is a clear need for research to identify optimal strategies for postpartum ART services for HIV-infected pregnant women and their exposed infants. To date, most research and programmatic attention to the implementation of PMTCT services has focused largely on the antenatal period, targeting the identification of HIV-infected women and/or the provision of PMTCT services during gestation and labour/delivery. Given the concerns around PMTCT services during the postpartum period, there is a need for greater attention to services and outcomes that include the complete PMTCT 'cascade', from entry into services through determination of infants' final infection status, in order to identify feasible and effective interventions to eliminate new paediatric infections and keep mothers healthy.

The overall aim of the proposed research is to evaluate two different strategies for delivering HIV care and treatment services during the postpartum period to eligible HIV-infected women who initiate antiretroviral treatment (ART) during pregnancy and their HIV-exposed infants. The study's primary objective is to compare an MCH-focused ART service to general adult ART services as strategies for providing ART during the postpartum period. The primary outcomes are (i) maternal HIV viral suppression, and (ii) maternal retention in ART services, at 12 months postpartum. Additional objectives focus on understanding the PMTCT 'cascade' (including the drop-out of ART-eligible women before delivery); investigating breastfeeding practices in the context of maternal ART use; MTCT rates at different times postpartum; and the acceptability and cost-effectiveness of the postpartum ART strategies under evaluation.

The study is to be conducted at the Gugulethu Midwife Obstetric Unit (MOU) in Cape Town. The study design has three interrelated phases.

- **Phase 1** is a cross-sectional evaluation of consecutive HIV-infected pregnant women seeking antenatal care at the study clinic (up to n=1600)

- **Phase 2** of the study is an observational cohort of all women from Phase 1 who are eligible for initiation of ART (up to n=600), followed in three study measurement visits, from their second antenatal clinic visit until their first postpartum clinic visit. ART initiation and antenatal follow-up takes place at the Gugulethu MOU according to standard provincial protocols.

- **Phase 3** of the study is a randomised trial of strategies for delivering ART to women during the postpartum period (the primary objective; n= up to 480). Women enrolled in Phase 2 who are breastfeeding their infants will be approached to participate in the trial at the end of the first routine postpartum clinic visit. Phase 3 will randomise women to one of two approaches to providing ART during the postpartum period to HIV-infected mothers who are breastfeeding:

  - <u>Arm A:</u> referral of women to their nearest general adult ART services at approximately 4-8 weeks postpartum (the current standard of care in this setting)

  - <u>Arm B:</u> continued receipt of ART in the antenatal clinic, as part of a MCH-focused ART service at the Gugulethu MOU that only refers women to general adult ART services after the end of breastfeeding and once infants' final HIV status is determined

  These two arms both employ standard provincial protocols for ART services (with identical medications and routine monitoring); they differ by the length of time women remain in the MCH-focused ART service at the Gugulethu MOU before they are referred to general ART clinics.

All women participating in Phase 3 will be followed through 18 months postpartum. The total length of participation in the study will vary based on gestational age at enrolment into Phase 2, ranging from a minimum of approximately 52 weeks to a maximum of approximately 80 weeks. Study measures will be taken at measurement visits that are conducted away from routine antenatal, postnatal or ART services (separate research staff working in a separate location). Measurements include questionnaires and HIV viral loads at each visit, as well as abstraction of clinical data on routine antenatal, postnatal and ART services received, for both mothers and infants.

Each Phase of the study has a separate Informed Consent (IC) process outlining the purpose and process of that Phase of the study, the risks and benefits of participation, the alternatives to participation, and that the decision to participate will in no way affect the medical care they receive. Participants may withdraw from the study at any time. Participants will receive up to R100 reimbursement to compensate for transport and time involved in attending the visit. They will be given R80 in grocery vouchers and R20 cash to cover transport for each visit. At the birth visit and the final study visit (at 18 months postpartum) they will also receive a small gift up to the value of R50 (eg, baby clothes or nappies).

Given the importance of MTCT in ART-eligible women, and the increasing importance of the postpartum period as a time of MTCT risk when breastfeeding, the proposed study has the potential to make a significant contribution to services that maximize the benefits of antiretroviral therapy for both maternal and child health.

1. Background

Over the last several decades there have been unprecedented advances in prevention of mother-to-child transmission of HIV infection (PMTCT). Clinical trials and observational studies have demonstrated safe and highly efficacious approaches to prevent HIV transmission using combination antiretroviral therapy (ART) during pregnancy, delivery and breast feeding [1]. At the same time, the global scale-up of HIV prevention, care and treatment services has resulted in a dramatic expansion of PMTCT and ART programs throughout the world, particularly in sub-Saharan Africa where more than 90% of HIV infected children currently reside [2]. Widespread use of combination ART in Europe and North America have resulted in transmission rates <1% [3-4]. Similarly, increased access to ART and PMTCT services in sub-Saharan Africa has also resulted in a substantial reduction in new paediatric infections: there were an estimated 330,000 new HIV infections among children globally in 2011, a 24% reduction since 2009 [2, 5].

There have been major advances in the provision of PMTCT services in South Africa in particular. Results of a representative national survey of infants attending immunization clinics during 2010 found early MTCT rates (determined by HIV PCR at 6 weeks of age) ranging from 3%-5%, a substantial achievement [6]. Preliminary results of a follow-on study conducted during 2011 suggest even lower 6-week transmission levels in many parts of the country, including the Western Cape [7]. MTCT rates after 6 weeks remain poorly understood, however, and are of major concern given recent shifts in South African PMTCT policy to the promotion of breastfeeding. Nonetheless, these and similar findings of decreased MTCT rates have propelled a South African plan towards elimination of new HIV infections in children by 2015 targeting a reduction in the number of new childhood HIV infections by 90% and the number of HIV-related maternal deaths by 50% [8-9].

While the successes to date in PMTCT in South Africa are encouraging, current approaches to PMTCT implementation across the country face significant challenges. Successful PMTCT interventions rely on use of antiretroviral drugs to suppress maternal viraemia and for prophylaxis to the fetus/newborn throughout the duration of exposure to maternal HIV, including during pregnancy, delivery and when breastfeeding. Providing effective PMTCT services, with ART when indicated, requires engagement in care and initiation of antiretroviral interventions either before or early in pregnancy [10-11]. Thereafter, mothers must be retained in care throughout pregnancy and the postpartum period of breastfeeding until a final infection status can be determined [12-13]. Throughout the duration of fetal/infant HIV exposure (from as early as possible in pregnancy through the end of breastfeeding), high levels of maternal adherence to ART are critical to reach and maintain maternal viral suppression, the most significant determinant of both transmission risk and long-term maternal health [14-15]. Yet there is growing concern that in the context of ART in pregnancy, women’s treatment adherence and retention in care may be highly problematic, particularly during the postpartum period [16-19], contributing to ongoing HIV transmission risk.

Key issues in optimizing ART for maternal and child health

The proposed research is based on recent developments in our understanding of both PMTCT and the delivery of ART services.

*Viral suppression drives MTCT transmission risk during pregnancy and postpartum***.** Most MTCT takes place in ART-eligible women who have the highest pre-ART viral loads: among HIV-infected pregnant women in Zambia, 77% of MTCT during the perinatal period as well as 83% of transmission in the postnatal period took place in ART-eligible women with CD4 cell counts<350 cells/µL [20]. Maternal viraemia is the critical determinant of MTCT *in utero*, intrapartum and postpartum, and the primary effect of antiretrovirals on MTCT is through reducing maternal viral load. Viral suppression at delivery and during the postpartum period is a direct function of pre-ART viral load and duration of ART [21]. There is good evidence from South Africa showing that increasing duration of ART in pregnancy is strongly associated with reduced infant transmission [22], with MTCT rates of 9.3%, 5.5% and 3.5% observed in ART-eligible women on ART for <4 weeks, 4-16 weeks, and > 16 weeks, respectively [11]. Other research has demonstrated that the benefits of early ART initiation extend to the postpartum when infants continue to be exposed to maternal HIV through breastfeeding [23].

Adherence to ART and retention in care are required to sustain viral suppression. High levels of treatment adherence are required to suppress viraemia in any patient using ART [14]. There is growing concern however that in some settings, women's adherence to ART either during pregnancy and/or during the postpartum period may be suboptimal, and contribute in turn to increased risk of vertical HIV transmission. In addition, retaining individuals in ART services is a necessary precursor to treatment adherence [24]: individuals who do not attend ART appointments (and do not collect their medications) are not adherent to their medication [25]. There is mounting evidence that failure to retain patients in care is the most widespread form of treatment non-adherence, and loss to follow-up from ART programmes is growing across the country [26]. Retention in care may be particularly problematic in women who initiate ART during pregnancy, leading directly to non-adherence, failure to suppress HIV viral loads, and ongoing transmission risk to exposed infants [27]. In addition, it is important to note that HIV-infected women with advanced disease are at a dramatically increased risk of maternal morbidity and mortality [28-29]; as a result, long-term retention in ART services is critical to sustaining maternal health over time [30].

Increasing postpartum HIV exposure in South Africa due to increasing breastfeeding. While there has been a long-running debate regarding the best strategy for infant feeding in the context of HIV infection, with the Tshwane Declaration of 2011 South African policy shifted to recommend breastfeeding for all HIV-exposed infants. Following from this, there have been dramatic shifts in breastfeeding practices among HIV-infected women, including in the Western Cape (where formula feeding had been encouraged for HIV-infected women). In women with advanced HIV disease, use of ART during the postpartum is the single most important strategy to allow the benefits of maternal breastmilk for the infant while minimizing transmission risk [31]. The best available evidence suggests that the vast majority of HIV-positive women in South African women who choose to breastfeed stop doing so by 6 months of age, though this may increase somewhat with new breastfeeding guidelines [32-33], pointing to the first 6 months of

the postpartum period as a critical window for interventions that can promote maternal ART adherence and retention in care.

Postnatal services for women on ART have received little attention. Multiple impediments to providing ART to eligible HIV-infected women have been described in South Africa and other settings. These include: health systems barriers (for example, transfers between clinics with multiple appointments for antenatal care and ART services, lack of prioritization within ART services compared with nonpregnant adults who have more advanced HIV disease) [34-35], biomedical barriers (delays in obtaining CD4 count and other laboratory results, complex ART regimens) [36], and patient-level barriers (stigma and disclosure, transport and other costs) [37-38]. There have been recent advances in South Africa to help address several of these factors. These include attention to the integration of ART services into antenatal care settings (for example, ART provided in the Midwife-Obstetric Unit (MOU) is routine in many parts of Cape Town); attention in national and provincial policies to ART-eligible pregnant women as a priority population that requires ‘fast tracking’ ART initiation; and the growing use of point-of-care (POC) CD4 count enumeration for pregnant women [39-40]. But most of these developments have targeted ART initiation during the antenatal period, and there are few service interventions for women on ART during the postnatal period.

In light of the above, there is a clear need for innovative operations research to identify optimal strategies for implementing ART services for HIV-infected pregnant women and their exposed infants. To date, most research and programmatic attention to the implementation of PMTCT services has focused largely on the antenatal period, targeting the identification of HIV-infected women and/or the provision of PMTCT services during gestation and labour/delivery. Yet given the concerns around PMTCT services during the postpartum period, there is a need for greater attention to services and outcomes that include the complete PMTCT ‘cascade’, from entry into services through determination of infants’ final infection status, in order to identify feasible and effective interventions to eliminate new paediatric infections and keep mothers healthy. The proposed study directly addresses this need.

2. Study aims & objectives

Study aim

The overall aim of this project is to evaluate two different strategies for delivering HIV care and treatment services during the postpartum period to eligible HIV-infected women who initiate antiretroviral treatment (ART) during pregnancy and their HIV-exposed infants.

Primary objective

1. To compare an MCH-focused ART service to general adult ART services as strategies for providing ART during the postpartum period on (i) maternal HIV viral suppression, and (ii) maternal retention in ART services, at 12 months postpartum.

Secondary objectives

2.1 To characterize the health status of the population of HIV+ pregnant women seeking antenatal care at the Gugulethu Midwife Obstetric Unit (MOU) including maternal and infant outcomes
2.2 To describe uptake and timing of services received throughout the PMTCT cascade, including antenatal ART initiation and follow-up, at the Gugulethu MOU
2.3 To examine whether there is variation in the impact of the two postpartum HIV care strategies on the primary outcome between subgroups of women according to their demographic, clinical and psychosocial characteristics, measured antenatally and postnatally.
2.4 To describe changes in women's clinical and/or psychosocial characteristics between the antenatal and postnatal periods, and investigate how these are independently associated with the primary outcome
2.5 To compare MCH-focused ART services to general adult ART services on:
   a. Women's missed visits and loss to follow-up on ART up to 18 months postpartum
   b. Breastfeeding practices, including duration of exclusive feeding and weaning patterns
   c. Maternal viral suppression throughout the breastfeeding period as well as before and after cessation of breastfeeding
   d. Infant health outcomes including growth parameters and incidence of respiratory and/or diarrhoeal illness
   e. Rates of HIV transmission from mother-to-child at 6 weeks and 12 months postpartum
   f. Acceptability of different strategies for delivering HIV care and treatment during the postpartum period
   g. Cost-effectiveness in delivering HIV care and treatment during the postpartum period
   h. Developmental scores of 1 year old infants

3. Study design

Overview

We propose to address these objectives using a three-phase design in which HIV-positive pregnant women are followed during the antenatal and postnatal periods. Throughout, participants will attend study measurement visits conducted separately from routine ART service appointments.

Phase 1 is a cross-sectional evaluation of consecutive HIV-infected pregnant women seeking antenatal care at the study clinic. This phase of the study will allow characterization of the health status of the population of HIV-positive pregnant women seeking care at the Gugulethu MOU and the services they receive (objectives 2.1 and 2.2). At their first antenatal clinic visit, women will be approached to complete Informed Consent #1, including consent for abstraction of routine clinic data on obstetric and HIV-related care. As part of this cross-sectional study, participating women will complete a short questionnaire and undergo phlebotomy.

Phase 2 of the study is an observational cohort of all women who are eligible for initiation of lifelong antiretroviral therapy (following local public sector guidelines [9]), from their second antenatal clinic visit until their first postpartum clinic visit (conducted within 7 days postpartum). This phase of the study will provide detailed description of ART initiation and antenatal follow-up in the population of women who will be involved in the postnatal component of the study

(objective 2.2), and be used to measure potential predictors of the primary outcome (objectives 2.3 and 2.4). At the second antenatal clinic visit when CD4 cell count and routine blood results are available, women from Phase 1 who have initiated ART or are ART-eligible will be approached to complete Informed Consent #2, be interviewed with questionnaires on behavioural and psychosocial measures, and undergo phlebotomy. Women will complete another study measurement visit during the third trimester and again within 1 week postpartum (with study measurement visits timed to coincide with routine care appointments). At these visits, additional questionnaires and phlebotomy will be used to collect study measures.

Phase 3 of the study is a randomised trial of strategies for delivering ART to women during the postpartum period (objective 1) with measurement of secondary outcomes during the postnatal period (objectives 2.3, 2.4, 2.5 and 2.6). Women enrolled in Phase 2 who are breastfeeding their infants will be approached to participate in the trial at the routine postpartum clinic visit (this takes place within 7 days of birth and is the only standard postpartum care for women). This coincides with the final Phase 2 study visit. Informed Consent #3 will be completed after completion of the Phase 2 postpartum visit. Randomization will take place after completion of Informed Consent #3.

Randomization will be to one of two approaches to providing ART during the postpartum period to HIV-infected mothers who are breastfeeding:

- **Arm A:** referral to general adult ART services from approximately 4-8 weeks postpartum (the local standard of care)
- **Arm B:** receipt of ART in the antenatal clinic, as part of a MCH-focused ART service that only refers women to general adult ART services after the end of breastfeeding and once infants' final HIV status is determined

Women randomized to Arm A or Arm B will be asked to return for five additional study visits during the postpartum period at approximately 6 weeks, 3 months, 6 months, 9 months, 12 months and 18 months postpartum. Details of study measurement visits conducted in both the antenatal and postnatal periods are described below in Section 8.

After completion of the12 months postpartum visit, women will be invited to participate in an additional infant neurodevelopmental assessment. This assessment is described in more detail in Section 8.

For women enrolled in Phases 1 and 2 of the study, participation will not impact on any aspect of routine antenatal and obstetric care during pregnancy. Similarly, throughout the antenatal period women's HIV-related care (including PMTCT services and ART) will not be affected in any way by participation, with ART initiated according to the current standard of care. For women enrolled in Phase 3 of the study, participation will only impact on the setting and approach to providing ART services to mothers during the postpartum period, comparing immediate referral to general adult ART services versus ongoing care in MCH-focused ART services within the Gugulethu MOU.

Overall, a total of approximately 1600 HIV+ pregnant women will participate in Phase 1 of the study over 12 months. From this group, it is anticipated that approximately 600 HIV+ pregnant

women eligible for ART will be identified as eligible for Phase 2 participation. We estimate that of the approximately 600 women on ART followed in Phase 2, approximately 480 will be eligible for and enrolled into Phase 3 and randomized to one of two postpartum ART delivery strategies.

Follow-up of all participants in Phase 3 will end at 18 months postpartum. The total length of participation will vary based on gestational age at enrolment into Phase 2, ranging from a minimum of approximately 52 weeks to a maximum of approximately 80 weeks. The study schema is summarized in the diagram below.

Figure 1: Study Schema

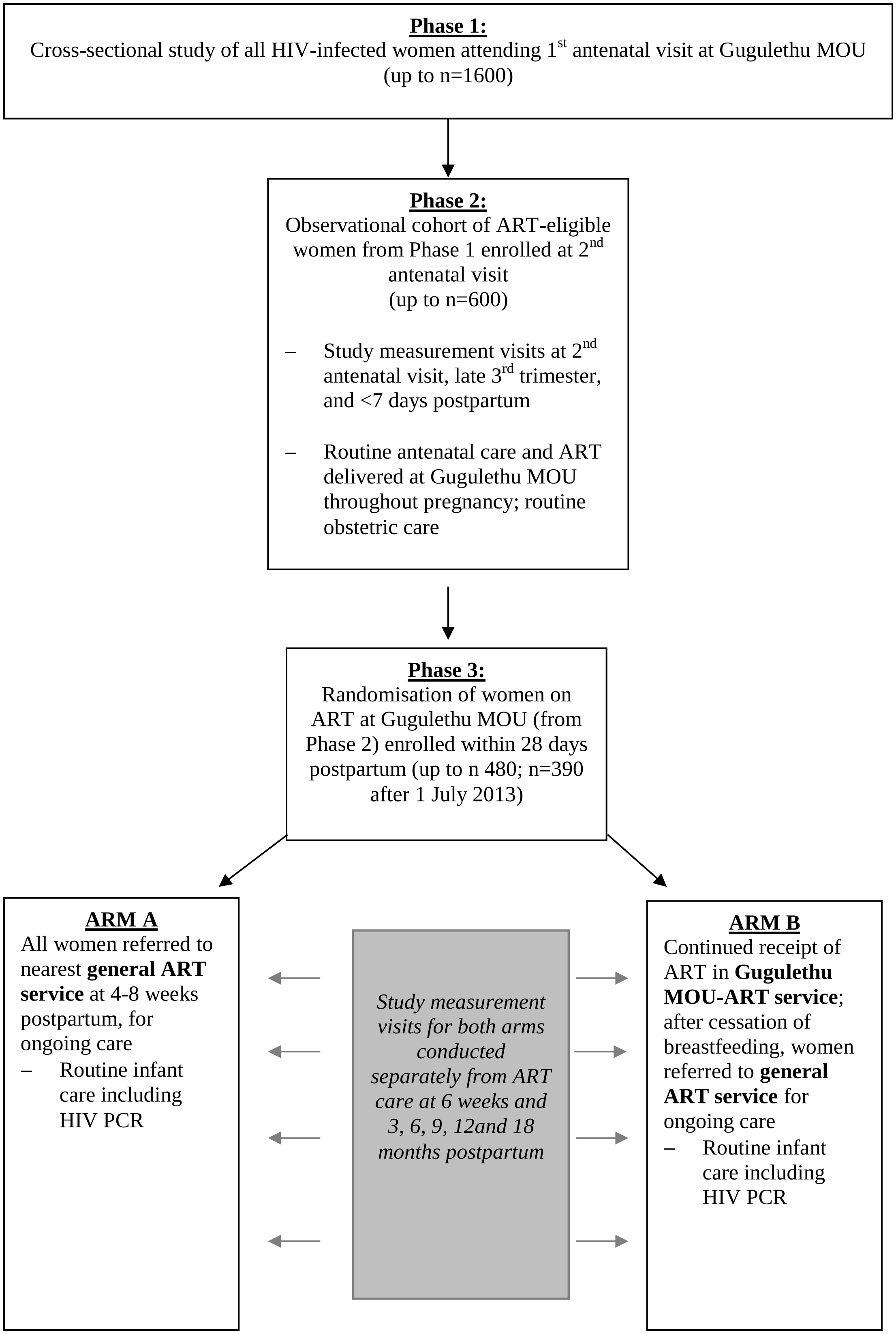

Setting

The proposed study will take place at the Gugulethu Community Health Centre (CHC) in Cape Town. Our team has helped to deliver HIV care and treatment services in this setting since 2003, and has a history of successful operations research on ART service delivery in partnership with the provincial government. In 2011, the HIV prevalence among women attending the Gugulethu ANC clinic was 31%, and 23% in the surrounding district. Primary care antenatal services are provided at the Gugulethu MOU. Approximately 4900 women sought antenatal care at the MOU in 2010. PMTCT services have been offered at the Gugulethu MOU since 2001; the MTCT rate (based on PCR at 6 weeks) is estimated at 2-4%. As part of the background to this research, our team has been working with the Gugulethu MOU services on issues related to ART initiation in pregnancy since early 2011.

4. Study population

Phase 1 participants will be consenting HIV+ pregnant women seeking antenatal care at the Gugulethu MOU, described above. Phase 2 participants will be the subset of Phase 1 participants who are ART-eligible. Phase 3 participants will be the subset of Phase 2 participants who elect to breastfeed their infants and who consent to be randomized postnatally to one of two postpartum HIV care strategies (Arm A vs. Arm B), described above. Women who complete the 12 months postpartum visit will also be invited to participate in an additional infant neurodevelopmental assessment.

Inclusion criteria

Phase 1

- Age 18 years or older
- Documented HIV-infection according to two finger-prick rapid tests using different test types (per routine protocol in this setting) or documentation of HIV status for those women self reporting HIV diagnosis.
- Confirmed pregnancy according to urine pregnancy test, ultrasound or clinical assessment
- Has not initiated triple-drug antiretroviral therapy or AZT for PMTCT during the current pregnancy
- Able to provide informed consent for research (Informed Consent #1)

Phase 2: Subset of Phase 1 participants who are ART-eligible

- Consented and participated in Phase 1
- Documented ART eligibility based on current local guidelines
- Started or scheduled to start ART at Gugulethu MOU in the current pregnancy (women started on AZT for PMTCT during the current pregnancy are eligible)
- Women who were previously receiving lifelong ART must have not used ART for at least 6 months.
- Able to provide informed consent for research (Informed Consent #2)

Phase 3: Subset of Phase 2 participants eligible for randomization

- Consented and participated in Phase 2
- Initiated ART during the antenatal period
- Currently breastfeeding within <7 days postpartum (with an allowable window of up to 28 days postpartum)
- Willingness to be randomized and return for postnatal study visits
- Able to provide informed consent for research (Informed Consent #3)

Infant neurodevelopmental assessment: Infants of women completing the 12 months postpartum visit
- Completed participation in Phase 3
- Infant does not have any major congenital anomaly
- Infant is not known to be HIV infected at age 12 months

Exclusion criteria

Individuals meeting any of the following exclusion criteria at the point in the study will be excluded:
- Not currently pregnant (Phases 1 and 2) or loss of pregnancy/neonate (Phase 3) at the time of eligibility determination
- Intention to relocate out of Cape Town permanently during the study period (Phase 2 and 3 only)
- Any medical, psychiatric or social condition which in the opinion of the investigators would affect the ability to consent and/or participate in the study (all phases), including:
    - Refusal to take ART/ARVs
    - Denial of HIV status

5. Recruitment

All women at the Gugulethu MOU making their first antenatal visit during the current pregnancy with documented HIV infection will be told about the study by MOU staff. If, after listening to the study introduction, women express interest in the study, MOU staff will have the option to (1) introduce them directly to the study staff based at the clinic or (2) request permission from the woman for study staff based at the clinic to approach them directly. For interested women, study staff will provide basic information about the study and, if interested in participating, women will be screened based on the inclusion/exclusion criteria listed in Section 4, above. Those women who are deemed eligible and agree to participate in Phase 1 will complete a written informed consent, Informed Consent #1, that includes consent to be approached for future research.

At the second antenatal clinic visit, Phase 1 participants who are ART- eligible and meet eligibility criteria listed in Section 4 will be approached by study staff to participate in Phase 2. Those who agree and are eligible for participation will complete a second written informed consent, Informed Consent #2. At the first postpartum clinic visit (less than 7 days), Phase 2 participants who meet eligibility criteria listed in Section 4 will be approached by study staff to participate in Phase 3. Those who agree and are eligible for participation will complete, Informed

Consent #3.Throughout, women who participate in Phase 1 (and complete Informed Consent #1), but who are not eligible for or refuse participation in Phases 2 or 3, will still have information abstracted from their routine clinical records.

After completion of the 12 months postpartum visit, Phase 3 participants who have infants that meet the criteria listed in Section 4 will be approached by study staff to participate in an additional infant neurodevelopmental assessment. Those who agree will complete a separate informed consent.

Procedures for the informed consent process are outlined in Section 9, below. Throughout, trained study staff will ensure that individuals are aware of their right to refuse and/or withdraw from the study at any time. In addition, study staff will emphasize that all study activities are entirely separate from routine antenatal and postnatal care services received and that refusal or withdrawal from the study will have no impact on their ability to access any antenatal services provided at any public sector health facility. Moreover, staff will be sure participants understand that their access to ART during pregnancy and postpartum, and any other aspect of their antenatal or postnatal care, will not be affected by their decision to participate.

6. Antenatal PMTCT and ART Services at the Gugulethu MOU

Phase 1 (cross-sectional study) and Phase 2 (cohort study) take place during the antenatal period, and will observe women receiving routine PMTCT and ART services at the Gugulethu MOU. All services will follow the local standard of care. Briefly:

- At the first ANC visit, all pregnant women undergo the following procedures:
    - routine blood testing (including ABO blood group, syphilis screening and haemoglobin) sent to the National Health Laboratory Services (NHLS) laboratory
    - medical and obstetric history and examination performed by a nurse-midwife
    - Rapid HIV testing, with pre/post-testing counselling
        - Women who are HIV-infected receive counselling, undergo venesection for serum creatinine and CD4 enumeration, and then women not already taking ART are started on a triple-drug antiretroviral regimen immediately, per local PMTCT guidelines.
        - Breastfeeding counselling begins at the first antenatal care visit, with all women encouraged to exclusively breastfeed for 6 months. Follow-up counselling is provided throughout the antenatal period.
        - Pregnant women receiving ART at the MOU return 1-2 monthly for follow-up visits and medication refills until delivery. Women already receiving ART elsewhere, continue their care at their current ART clinic.
- Labour and delivery take place at either the Gugulethu MOU or nearby obstetric hospitals. All women make a routine postnatal visit to the Gugulethu MOU within 7 days postpartum; this is the only form of maternal postnatal care in this setting.

7. Postnatal ART Services

For Phase 3 participants, postnatal ART services will compare the following ART delivery strategies:

- **Arm A:** the current standard of care defined as referral of women on ART and their HIV-exposed infants to general adult ART services at 4-8 weeks postpartum;
- **Arm B:** MCH-focused ART services defined as retention of women on ART in the MOU-based service throughout the period of breastfeeding, with ongoing counselling and support on maternal and child health and PMTCT, as well as routine infant care (including EPI vaccinations, growth monitoring and early infant HIV diagnosis) provided in the MOU-based service.

<u>General adult ART management strategy</u>
Women and infants randomized to the general adult ART management strategy will receive postpartum ART services according to the local standard of care. Specifically, women will be referred from the MOU ART service to their nearest adult ART clinic at their first postpartum ART clinic visit, at around 6 weeks postpartum. The precise timing of this referral will depend on the scheduling of women's ART visits, and may take place between approximately 4 and 8 weeks after birth. Infants will be referred for routine infant care to local primary care clinics, following standard of care in this setting.

The specific facility women are referred to will be determined by their area of residence. For women who reside within Gugulethu itself, referral will be to the ART service at the Gugulethu CHC (located approximately 100 metres from the MOU; this applies to approximately 60-70% of women attending the MOU); the corresponding infant referral is to one of four primary care clinics in the local community. For women who live in other communities (with the Gugulethu MOU as their closest antenatal clinic, but not their closest ART clinic), referral will be to their nearest adult ART clinic, primarily facilities serving the communities of Philippi and Nyanga. All infants will be referred to their nearest routine infant follow-up service. Women may be referred to other facilities (e.g., in other parts of Cape Town or in the Eastern Cape) upon request.

In addition, midwives working in the MOU-ART service may opt to refer a woman to a higher level of medical care (e.g., if there is concern regarding ART toxicity) or on the basis of clinical or psychosocial grounds (if there are concerns regarding non-adherence). Reasons for deferred or alternate referral are documented in the clinical record.

On the day of referral, all women receive counselling on long-term ART adherence, the ongoing risks of MTCT through breastfeeding, and the need for repeat infant HIV testing. Following provincial protocols, referrals employ a Western Cape ART transfer form, completed in duplicate. The original form is sent with the patient, and the copy is retained in the patient's MOU folder. Patients are prescribed one month of treatment and advised to make an appointment at their referral clinic as soon as possible before their one-month ART supply is finished.

Following transfer to routine ART care, postpartum women are incorporated into the general population of adults receiving ART (including transfers in from other ART services). At the first

appointment after transfer into the general ART service, patients undergo clinical history and examination by a doctor or clinical nurse practitioner. Laboratory investigations are ordered according to standard protocols or based on clinician discretion. At this first visit in the new service, stable patients are dispensed 1-2 months of ART; subsequent visits are 1-2 monthly for medication refill, with clinician review 6-monthly; patients with particular clinical or psychosocial concerns may be reviewed more regularly, or referred to higher levels of care. Throughout, clinical care is provided according to the same SA national protocols and using the same records as used in the MOU-ART service.

<u>MCH-focused ART management strategy</u>
Women randomised to the MCH-focused ART management strategy will be retained in the MOU-ART service, along with their infants, throughout the period of breastfeeding, and referred to their nearest adult ART clinic (as described above) only after the end of breastfeeding. ART visits will be scheduled 1-2 monthly throughout this period. At each clinic visit during the postpartum period, mothers and infants will receive:

- Routine ART care, following local protocols
- Review of infant feeding practices specifically
- Routine infant health care (including infant weight monitoring, vaccination and early infant HIV diagnosis) with completion of the infant's Road-to-Health Card. This is the identical set of services to those provided through local primary care clinics; in all instances, there is referral to local paediatric services when required
- Ongoing PMTCT counselling, including messaging on ongoing transmission risk during the postpartum period; ART adherence; safe infant feeding practices; adherence to daily nevirapine prophylaxis (through 6 weeks postpartum per local guidelines); and the need for infant HIV testing at 6 weeks postpartum and again after cessation of breastfeeding

At each postpartum visit, mothers will be asked basic questions on infant feeding practices related to current feeding practice, plans for weaning and future feeding intentions. Mothers who are found to have not breastfed for at least 1 week before a clinic visit, and who do not state an intention to resume breastfeeding, will be eligible for referral out of the MOU ART service. Referrals out of the MOU ART service will follow the guidelines described above. For mothers who are referred out of the MOU, infants will be referred to their nearest routine infant follow-up service.

<u>Infant follow-up</u>
Infant follow-up procedures are identical for women in Arm A or B, but the location of infant follow-up differs (either in the MOU for infants whose mothers are retained postpartum in the MOU, or at the nearest primary care clinic for infants of mothers who have been referred out). Following national protocols, all HIV-infected mothers are issued a 6-week supply of nevirapine syrup after delivery and counselled on daily nevirapine prophylaxis. Infant follow-up takes place within 1 week postpartum with the mother (at the MOU) and then at 6 weeks postpartum (either at the MOU or the nearest primary care clinic) when HIV PCR testing of the infant is carried out. Following national protocols, infants who are breastfed beyond 6 weeks (including all infants of mothers participating in Phase 3 of the study) receive HIV PCR testing 2-4 weeks after the cessation of breastfeeding, again conducted either at the MOU or at the nearest City of Cape Town primary care clinic.

8. Study Procedures

The purpose of the study measurement visits will be to evaluate the primary and secondary study objectives. These **study measurement visits** will be carried out separately from antenatal care and ART services throughout the research. For women participating in all three phases of the study, a maximum of nine visits may take place from the beginning of antenatal care through 18 months postpartum. The schedule of study measurement visits is shown in the table below.

Phase 1

Following recruitment and informed consent at the first antenatal clinic visit, women who participate in Phase 1 will complete a brief study measurement visit (*study visit 1*; approximate duration, 20 minutes total).

- A short standardized questionnaire will be used to collect information on: demographic characteristics, socioeconomic status, medical history, family planning, HIV testing history and disclosure status, and previous antiretroviral exposure. Additional information related to HIV treatment knowledge, beliefs about ART medications and ART adherence will be collected from women already on ART enrolling into Phase 1.
- Participants will undergo phlebotomy to draw 5mL of venous blood for batched viral load testing.
- The informed consent for Phase 1 includes consent to abstract clinical data from patient folders and PMTCT/MOU registers and permission to be contacted to participate in further research.

Phase 2

Participants who are eligible for ART according to current local recommendations for ART eligibility, and who meet other eligibility criteria outlined in Section 4, will be invited to participate in Phase 2 of the study at the end of their second antenatal clinic visit. As part of this, women completing informed consent #2 will complete three study measurement visits: on the same day (i.e., after the second antenatal clinic visit, *study visit 2*), during the late third trimester (*study visit 3*; scheduled based on gestation at enrolment into Phase 2 to be approximately 32-36 weeks' gestation), and within 7 days postpartum (*study visit 4*; timed to coincide with the routine postnatal clinic visit held 2-4 days postpartum). These study measurement visits will last 30-45 minutes each. At these visits:

- *Antenatal study visits*. Questionnaires will be administered on recent pregnancy- and HIV-related health care, HIV disclosure, and ART use (including side effects and adherence). At selected antenatal visits, additional instruments will collect data on HIV treatment knowledge, beliefs about ART medications, stigmatization, social support, experiences of partner violence, and mental health measures.
- *Postpartum study visit*. Questionnaires will be administered on antenatal, obstetric and HIV-related health care, HIV disclosure, and ART use (including side effects and adherence). Additional instruments will collect data on obstetric information, family planning, quality of HIV care during pregnancy, infant feeding practices, and infant health and health care (including adherence to daily nevirapine prophylaxis).

- At each study visit, participants will undergo phlebotomy to draw 5mL of venous blood for storage and batched viral load testing.

Phase 3

Women completing the postpartum study measurement visit (*study visit 4*) of Phase 2 will be invited to participate in Phase 3 of the study. Women will be screened for eligibility (including breastfeeding) and complete Informed Consent #3. Consenting women will be randomized to either be referred to general adult ART services at their next scheduled visit to the MOU-ART service (at approximately 4-8 weeks postpartum) or to continue receipt of ART in the MOU clinic, as part of a MCH-focused ART service that only refers women to general adult ART services after the end of breastfeeding and once infants' final HIV status is determined. Infants' allocation will follow those of their mothers, to either receive routine infant care at the nearest primary care clinic, or in the MOU.

Randomization

Randomization will take place at the end of the study measurement visit within the first week postpartum. Randomization will follow a dynamic permuted block design. Randomization numbers will be generated prior to the start of the study, and placed in sequentially numbered opaque envelopes. Randomization envelopes will be stored in a locked cabinet in the study office at UCT that will be accessed by the study coordinator when a woman is fully consented and has completed the immediate postpartum study visit, with independent documentation of the participant ID number, randomization date, and randomization assignment. The study coordinator will provide the MOU-ART service with a regularly updated list of women to be retained within the MOU-ART service until they have ended breastfeeding. The study coordinator involved in randomization will not be involved in interviewing participants.

Six week postpartum study visit *(study visit 5)*

Mother-infant pairs in both trial arms will be seen at 6-8 weeks postpartum. At this visit (lasting 30-45 minutes):

- Questionnaires will be administered on recent maternal, child and HIV-related health care, HIV disclosure, and ART use (including side effects and adherence). Additional instruments will collect data on HIV treatment knowledge, beliefs about ART medications, stigmatization, and mental health measures (including psychological distress, and perinatal mood), infant feeding practices, and infant health and health care (including adherence to daily nevirapine prophylaxis).
- Mothers will undergo phlebotomy to draw 5mL of venous blood for storage and batched viral load testing.
- Mothers height, weight and mid-upper arm circumference will be measured
- Growth parameters of the infant will be measured (weight, length, head circumference and mid-upper arm circumference)

Three month postpartum study visit (study visit 6)

Mother-infant pairs in both trial arms will be seen at 3 months postpartum. At this visit (lasting 30-45 minutes):

- Questionnaires will be administered on recent maternal, child and HIV-related health care, HIV disclosure, and ART use (including side effects and adherence). Additional instruments will collect data on infant feeding practices and infant health and health care.
- Mothers will undergo phlebotomy to draw 5mL of venous blood for storage and batched viral load testing.
- Mothers weight and mid-upper arm circumference
- Growth parameters of the infant will be measured (weight, length, head circumference and mid-upper arm circumference)

Six month postpartum study visit *(study visit 7)*
Mother-infant pairs in both trial arms will be seen at 6 months postpartum. At this visit (lasting 30-45 minutes):

- Questionnaires will be administered on recent maternal, child and HIV-related health care, HIV disclosure, ART use (including side effects and adherence) and quality of life. Additional instruments will collect data on quality of HIV care, maternal family planning, future pregnancy intentions, and mental health measures (including alcohol/substance abuse), infant feeding practices, and infant health and health care.
- Mothers will undergo phlebotomy to draw 5mL of venous blood for storage and batched viral load testing.
- Mothers weight and mid-upper arm circumference will be measured
- Growth parameters of the infant will be measured (weight, length, head circumference and mid-upper arm circumference)

Nine month postpartum visit *(study visit 8)*
Mother-infant pairs in both trial arms will be seen at 9 months postpartum. At this visit (lasting 30-45 minutes):

- Questionnaires will be administered on recent maternal, child and HIV-related health care, HIV disclosure, and ART use (including side effects and adherence). Additional instruments will collect data, maternal family planning, future pregnancy intentions, and mental health measures, infant feeding practices, and infant health and health care.
- Mothers will undergo phlebotomy to draw 5mL of venous blood for storage and batched viral load testing.
- Mothers weight and mid-upper arm circumference will be measured
- Growth parameters of the infant will be measured (weight, length, head circumference and mid-upper arm circumference)

Twelve month postpartum visit *(study visit 9)*
Mother-infant pairs in both trial arms will be seen at 12 months postpartum. At this visit (lasting 60 minutes):

- Questionnaires will be administered on recent maternal, child and HIV-related health care, HIV disclosure, ART use (including side effects and adherence) and quality of life. Additional instruments will collect data on quality of HIV care, maternal family planning, partner violence, future pregnancy intentions, and mental health measures (including alcohol/substance abuse), infant feeding practices, food security and infant health and health care

- Mothers will undergo phlebotomy to draw 5mL of venous blood for storage and batched viral load testing.
- Mothers weight and mid-upper arm circumference will be measured
- Growth parameters of the infant will be measured (weight, length, head circumference and mid-upper arm circumference)
- Infants will undergo phlebotomy. No more than 5mL of venous blood will be drawn for HIV PCR testing with storage of remaining specimen. Results of this HIV testing will be returned to mothers by study staff, with urgent recall of any infant found to be infected for referral to clinical care. This blood will also be used to conduct a rapid antibody test for research purposes.
- After completion of above study procedures, mothers pairs will be invited to participate in an additional infant neurodevelopmental assessment. Infants will be screened for eligibility based on the criteria outlined in Section 4. If eligible, mothers will complete a separate written informed consent form. Those who consent to the additional assessment will be given the option to complete the neurodevelopmental assessment immediately (on the same day as 12 months postpartum visit) or be scheduled for an additional visit to take place within 4 weeks from the 1 year birthday of your baby.

Infant neurodevelopmental assessment *(study visit 10)*
After completion of the 12-months postpartum visit, all mother-infant pairs will be offered the opportunity to take part in this additional neurodevelopmental assessment. Interested women will sign a separate consent for these procedures.

- Mothers who consent to this additional infant assessment will undergo a single developmental assessment, lasting approximately 60-90 minutes. The Bayley Scales of Infant and Toddler Development 3rd edition (BSID-III) will be administered by a trained study doctor or occupational therapist. The assessments will be conducted in a dedicated, quiet, child-friendly space, with the assistance of a trained clinical research assistant fluent in both English and isiXhosa.

- Infants will be assessed on all five domains, namely cognitive, language, motor, adaptive behaviour and socio-emotional. Adaptive behaviour and socio-emotional testing will be conducted in maternal interview form, using approved isiXhosa translated versions of the BSID-III report forms.

- Infants will be provided with a snack approved by the mother prior to assessment, and the session will be interrupted for a short break of 5-10 minutes if the child shows any sign of distress such as crying, whining or refusal. In the event of intractable infant distress or current illness, assessments will be postponed with the mother’s agreement.

Eighteen month postpartum visit *(study visit 11)*
Mother-infant pairs in both trial arms will be seen at 18 months postpartum. At this visit (lasting 60 minutes):

- Questionnaires will be administered on recent maternal, child and HIV-related health care, HIV disclosure and ART use (including side effects and adherence). Additional instruments will collect data on maternal family planning, future pregnancy intentions, and mental health measures, infant feeding practices, and infant health and health care.

- Mothers will undergo phlebotomy to draw 5mL of venous blood for storage and batched viral load testing.
- Mothers weight and mid-upper arm circumference will be measured
- Growth parameters of the infant will be measured (weight, length, head circumference and mid-upper arm circumference)
- Infants who are not known to be HIV-infected will undergo phlebotomy. No more than 5mL of venous blood will be drawn and used to conduct a rapid antibody test. For those infants who are reactive via antibody test, additional HIV PCR testing will be conducted with storage of remaining specimen. Results of this HIV testing will be returned to mothers by study staff, with urgent recall of any infant found to be infected for referral to clinical care.

Verbal autopsy
In the event of the death of an infant in the study, the World Health Organisation standard verbal autopsy for investigating causes of death in infants and children will be administered to collect information on possible cause of death.

Laboratory measures
Laboratory specimens taken for this research include maternal venous blood (approximately 5 mL at up to ten study visits, or a maximum of approximately 50mL of blood over a maximum of 80 weeks including antenatal and postnatal follow-up) and infant venous blood (no more than5mL at 12 and 18 months postpartum).

At each study measurement visit, a maternal specimen will be taken in SST tubes and transported to NHLS for storage. Maternal specimens will be used for HIV viral load testing (Abbott Molecular RealTime HIV-1 assay (Abbott Molecular, Illinois, USA), done in batch testing. The remaining plasma will be divided into 3-4 aliquots of ±80µL each and stored for future research in Sarstedt screw cap tubes at -80°C.

Infant bloods collected at 12 and 18 months will be analyzed at NHLS using the Roche Cobas AmpliPrep/Cobas TaqMan HIV-1 qualitative assay. At 18 months, only infants who test positive via rapid antibody test will be have subsequent PCR testing conducted. Results will be returned to study personnel to notify mothers of test results immediately; any infant found to be HIV-infected at any time during the study will be recalled telephonically and/or by home visit for urgent counselling and referral to care. The remaining plasma will be divided into 2-4 aliquots and stored for future research in Sarstedt screw cap tubes at -80°C.

All phlebotomy, specimen handling, specimen processing, and specimen storage will take place using routine protocols. Note that the laboratory measures taken for study measurements are in addition to routine laboratory tests taken for antenatal care or HIV care and treatment. For all women receiving ART, participants will have laboratory measures following national ART protocols including ART safety bloods (eg, creatinine for estimation of creatinine clearance) and ART monitoring (including CD4 and viral load testing) carried out in the either the MOU-ART or general adult ART service.

Abstraction of routinely collected clinical information

In addition to the study measures described above, chart reviews will be conducted in order to collect key data on participants regarding (a) antenatal and obstetric care and (b) ART initiation and follow-up. Permission to review clinical records of participants is included in informed consent documents for all participants.

- *Antenatal information* will be taken from MOU records, including the results of routine bloods (syphilis screening, ABO blood group), the number and timing of ANC visits, the results of prenatal ultrasound scan, and any evidence of antenatal complications including episodes requiring referral to specialist obstetric care.
- *PMTCT information*. Will come from antenatal and obstetric records, including: CD4 enumeration, dates and types of antiretroviral drugs received.
- *Obstetric information* will be abstracted from the records at the MOU or nearby obstetric hospitals. We will abstract the date and time of the onset of labour and rupture of membranes, the progress of labour and evidence of fetal distress, the date, time and mode of delivery; birth outcome; any PMTCT interventions received; Apgar scores at 1 and 5 minutes postpartum; neonate mass, head circumference and length, and placental weight.
- *ART initiation and follow-up data* will be abstracted at the end of the study from routine clinical care records maintained by the general ART and MOU-ART services. Items to be abstracted from these clinical records include: date of initial presentation to the ART service; pre-ART clinical history, examination and laboratory investigations (including TB-related history and WHO staging); date of ART initiation; dates of routine follow-up visits post-ART initiation and major clinical events, including clinical findings that may suggest opportunistic infections, ART side effects, drug toxicities; pharmacy dispensing & refill information; and missed follow-up visits and retention in care. Information through 18 months postpartum will be abstracted on all participants from the relevant clinical records at either the general ART or the MOU-ART service, as appropriate.
- *Infant information* will include information on routine 6-week HIV PCR testing (date, results); repeat PCR testing after the cessation of breastfeeding; infant immunizations; infant growth, and the nature, duration and management of any infant illnesses. Sources of data for this will be the Road-to-Health Card (for all infants) and hospital records (for infants admitted to hospital).

All data abstraction will take place with the written permission of (a) the participant (via informed consent), (b) the research oversight body of the Provincial Government of the Western Cape (PGWC), (c) the participating IRB/REC, and (d) the facility managers of each participating health facility. Data abstraction will be conducted by trained staff working under the close supervision of the project coordinator. All data are abstracted from the clinical record onto separate forms that include record of the type of documentation, the date of abstraction, and the name of the individual abstracting data. All data collection is confidential (with records identified via participant folder numbers only) and no participant names are recorded on study documents.

Schedule of study measurements

	**Phase 1** *(all HIV+ pregnant women)*	**Phase 2** *(HIV+ pregnant women eligible for ART)*			**Phase 3** *(HIV+ postpartum women on ART and breastfeeding)*					
Item for completion	**1st antenatal visit (visit 1)**	**2nd antenatal visit (visit 2)**	**Late 3rd trimester (visit 3)**	**< 7 days pp (up to 28 days) (visit 4)**	**6 weeks pp (visit 5)**	**3 months pp (visit 6)**	**6 months pp (visit 7)**	**9 months pp (visit 8)**	**12 months pp (visit 9)**	**18 months pp (visit 11)**
**Procedural forms**										
Eligibility Checklist	X	X		X						
Informed Consent	#1	#2		#3					Infant Neurodevelopmental Assessment ICF	
Locator Information [1]	X	X	X	X	X	X	X	X		X
Randomization Log				X						
**Questionnaires**										
Demographics & medical history	X	X[3]	X[3]	X[3]	X[3]	X[3]	X[3]	X[3]	X[3]	X[3]
HIV Knowledge Inventory	X[2]	X			X			X		
Treatment Knowledge Inventory	X[2]	X			X			X		
ART Medication Beliefs	X[2]	X			X			X		
Adherence self-efficacy	X[2]	X	X				X		X	X
K-10 & EPDS [4]		X			X				X	X
Alcohol/substance use screen [5]		X	X				X		X	
Trauma/abuse assessment		X		X					X	
Social impact scale		X			X				X	
Availability of social support scale		X	X				X		X	
Infant feeding intentions/practices			X	X	X	X	X	X	X	X
Family planning use/intentions	X			X	X		X	X	X	X
Pregnancy intentions	X			X	X		X	X	X	X
Patient-Provider relationship scale			X	X	X		X	X	X	X
Unplanned Pregnancy assessment	X									
Maternal adherence [6]	X[2]	X	X	X	X	X	X	X	X	X
Infant adherence[6]				X	X	X	X	X	X	X
Child Grants					X		X		X	
Infant demographics & medical history				X	X	X	X	X	X	X
Resource Interview							X		X	
Food security									X	
**Anthropometry**										
Maternal anthropometry [7]					X	X	X	X	X	X

	**Phase 1** *(all HIV+ pregnant women)*	**Phase 2** *(HIV+ pregnant women eligible for ART)*			**Phase 3** *(HIV+ postpartum women on ART and breastfeeding)*					
Item for completion	**1st antenatal visit (visit 1)**	**2nd antenatal visit (visit 2)**	**Late 3rd trimester (visit 3)**	**< 7 days pp (up to 28 days) (visit 4)**	**6 weeks pp (visit 5)**	**3 months pp (visit 6)**	**6 months pp (visit 7)**	**9 months pp (visit 8)**	**12 months pp (visit 9)**	**18 months pp (visit 11)**
Infant growth parameters					X	X	X	X	X	X
**Neurodevelopmental assessment**										
Bayley Scales of Infant and Toddler Development									X	
**Study laboratory measures**										
HIV viral load	X	X	X	X	X		X	X	X	X
Maternal stored serum	X	X	X	X	X		X	X	X	X
Infant HIV PCR testing									X	X[10]
Infant rapid antibody test									X	X
**Clinical data abstraction** [8]										
ART initiation & follow-up		X	X	X	X	X	X		X	X
Antenatal & obstetric information	X	X	X	X						
Pharmacy ART dispensing records		X	X	X	X	X	X	X	X	X
Maternal laboratory test results [9]	X	X					X		X	X
Infant growth and well-being				X	X	X	X	X	X	X
Infant HIV PCR result [9]					X		X			

1. Participants' locator information will be updated at each study visit, if it has changed since the last visit.
2. These assessments will be administered to participants who are already on ART at the time of enrolment into Phase 1.
3. A subset of demographic questions will be asked at all antenatal and postnatal study visits.
4. Kessler-10 (screening questionnaire for non-specific psychological distress) and Edinburgh Postnatal Depression Survey.
5. Alcohol use disorders identification test (AUDIT) and drug use disorders identification test (DUDIT).
6. Adherence assessments will include maternal ART adherence and, postpartum, questions related to infant adherence nevirapine prophylaxis and co-trimoxazole.
7. Note that maternal weight & mid-upper arm circumference will be measured at each Phase 3 study visit; maternal height will only be measured at the 6 week postpartum visit
8. Abstraction of routine clinical data and laboratory data will take place at the end of the study.
9. Results of routine laboratory testing on mothers (including pre-ART and on-ART CD4 cell count, pre-ART serum creatinine, alanine aminotransferase and full blood count) and infants (HIV PCR at 6 weeks and 2 weeks after cessation of breastfeeding; here marked for the 6 month visit) will come from record review.
10. 10. PCR testing will only be conducted on infants who are positive per rapid antibody test

Qualitative Component

In-depth Interviews

We will conduct qualitative in-depth interviews with a subset of up to 65 participants from Phase 3 of the study. Each woman will be interviewed one time. Interviews will be conducted by a trained isiXhosa-speaking research assistant using an interview guide in order to examine experiences including: study participation; ART initiation during pregnancy generally; post-partum follow-up on ART; and transitions to routine adult ART services. Qualitative interview participants will be selected purposively, to include women who are approximately 6-9 months post-partum. All participants will sign a separate informed consent form for this component of the study.

Clinic observation

Clinic observation in this study will consist of approximately five half-day sessions in the ART clinic within the MOU as well as the general adult ART clinic at Hannan Crusad and potentially another ART clinic location (to be identified), and will be conducted by the primary qualitative researcher, as well as one fieldworker. During clinic observation, the researchers will record extensive notes in the form of field notes, about the clinic environment. Informal conversations with both patients and staff will be recorded collectively. Field notes by both the primary qualitative researcher, as well as the field worker, will be read and discussed.

Cost-effectiveness

Costing data will be used alongside findings on clinical outcomes in order to understand the cost-effectiveness of the two strategies for ART services during the postpartum period. Data on unit costs, including human resources costs, will be abstracted from routine facility accounts, facility utilization data and staffing plans, and pharmacy accounts maintained by the PGWC; data on service utilization by participants will come directly from the study data; program costs will be based on local standards for training and materials development; overhead costs will be based on PGWC accounts.

Identification of health care needs during study procedures

At every study measurement visit, any woman found to have an unmet health need (whether medical, obstetric, postpartum family planning, or related to mental health or substance use) will be referred to the relevant service within the Gugulethu CHC or higher levels of care, as appropriate. In particular:

- Any woman found to have defaulted ART during any Phase of the study will be re-referred to the relevant ART service (general adult or MCH-focused) via the Gugulethu MOU.
- Any woman found to be non-adherent to ART at any study measurement visit will receive intensive counselling on ART use, and referred to the relevant ART service for follow-up.
- Any woman found to be experiencing domestic or partner violence in any form will be referred to the main local NGO supporting victims of domestic violence (MOSAIC) and the South African Police Service (per the Domestic Violence Act, No. 116 of 1998) with follow-up to ensure adequate attention.

- Any child found to be HIV-infected will be referred immediately to the nearest paediatric ART service for assessment and infant ART initiation.
- If any infant health care needs identified, the infant will be referred to the appropriate paediatric health services via the Gugulethu Community Health Centre.

Staff training

Prior to initiation of the study, all staff that will have contact with participants will take part in a multi-day study-specific training. The curriculum of the training will include: rationale, purpose, and scientific objectives of the study; study design and methodology; conduct of study assessments, tracking of participants, completion of study forms, and data collection; staff responsibilities; recruiting participants; procedures for enrolling participants into the study; randomization, universal precautions, communication skills, safety in the field, ethical guidelines for research including participants' rights; procedures for obtaining informed consent; and confidentiality requirements.

Study staff will receive a hands-on training that will include an introduction to data collection forms and procedures. Mock interviews will be an essential component of the training and protocol team members will act as both the trainer and the mock respondent. The trainer will take the staff through each step of the data collection process, from enrolling participants to ending the study visit and completing any necessary forms. Study staff will be given a chance to practice both the English and isiXhosa versions of all the assessments in order to discuss and resolve any issues. Training for study staff is expected to take approximately 1 week and additional follow-up training, as necessary.

Study staff members who will collect blood samples and conduct laboratory analyses will receive training in universal precautions, sample collection, and testing of study samples.

In addition, staff will be trained in the management of crisis situations, including reports of abuse and domestic violence,that may be disclosed during study participation. The site will have established procedures for managing these situations and procedures in place for providing counselling and appropriate referrals. Procedures for managing these issues will be outlined in study SOPs.

For all study staff, there will be additional training days scheduled during the study for refresher training. During these refresher trainings, study staff will review study procedures and discuss any challenges encountered. All staff who through the course of their work have knowledge of, or access to, personal information about participants will be required to complete training on patient confidentiality and sign a confidentiality agreement before the start of data collection.

Contamination and masking

Contamination between groups is a concern and blinding participants to this type of health systems evaluation is not possible. Contamination would mean that women in the general adult ART group may be aware of the MCH-focused ART group, or vice-versa, and change their behaviours related to their own ART use, retention in care, and/or infant feeding practices. Detection biases are possible for self reported outcomes due to lack of blinding, but one of the primary outcomes (HIV viral load) is objective. Insofar as possible, we will mask study

personnel involved in the study measurement visits. In addition, we will conduct the study measurement visits from a space at the Gugulethu CHC that is separate from both the general adult ART services and the MCH-focused ART services, with study staff who are different from participants' service provider, in order to minimize possible biases in assessment.

9. Participant Retention

At enrolment into Phase 1, all women will be asked to provide detailed locator information. Specifically, participants will be asked to provide the following information: full name, address, contact numbers (including landline and cellphone) and the names, addresses, and contact details for two individuals who the participant lives with and one alternate individual who the participant does not live with. At later visits, participants will be asked to update their contact information if needed. All locator information is kept in locked study cabinets accessible only under the direction of the study coordinator.

After enrolment, all study activities will take place through appointments conducted separately from ANC and ART care. There will be two main approaches to tracing participants who miss study appointments:

- Telephonic contact is made with the participant or with an alternate individual pre-designated by the participant.
- If telephone attempts fail, study fieldworkers visit the home address of the participant to trace the participant in person.

All participant tracing efforts by any staff (via phone or home visit) will not mention the reason for the contact, or anything regarding HIV/AIDS or ART. All contacts simply request that the participant come to the clinic the next working day for a health-related issue. Our research team has been working in this setting for more than 8 years, has close knowledge of the local community, and has extensive experience with retention activities around HIV/AIDS.

As part of research activities, we will include an assessment of whether the participant is taking ART. For those women found to be not using ART, staff will facilitate an urgent referral back to the appropriate services per randomization assignment. Details and examples of these retention procedures, including this adherence assessment, will be included in the staff training to be conducted prior to study implementation.

Participant withdrawal

All participants may refuse or voluntarily withdraw from the study for any reason and at any time. As part of the informed consent process, staff will state specifically that participation in the study is voluntary and that a participant may refuse participation or withdraw from study participation at any time. Participants will be told that withdrawal from the study will have no effect on their access to health facilities providing antenatal care or HIV-related testing, care and treatment services. All study staff will be trained to ensure that participants have a firm understanding of this concept at the time of the informed consent process; per consenting requirements, the informed consent form will also include a statement to this effect.

10. Analytic considerations

The study is a multiphase evaluation, with the main component designed to compare the impact of two different ART management strategies on clinical, behavioural, acceptability and cost-effectiveness outcomes among HIV- positive ART-eligible pregnant women seeking antenatal care. The primary outcomes, to be assessed using a randomised trial design (Phase 3), are viral suppression and retention in care of mother and infant at 12 months postpartum. The study analysis is driven by two competing hypotheses:

- ***Hypothesis 1***: Maintaining women in MCH-focused ART services during the postpartum period will result in a higher proportion of women retained in care and virally suppressed throughout the period of infant HIV exposure via breastfeeding, compared to referral of women to general adult ART services early postpartum.

- ***Hypothesis 2***: Referral of women to general adult ART services earlier in the postpartum period will result in higher levels of retention on ART at 12 months postpartum, compared to maintaining women in MCH-focused ART services throughout the breastfeeding period.

Sample size considerations

The sample size calculations are based on the following assumptions:

- A superiority comparison between the MCH-focused ART services group and the general adult ART services group using 90% power (β=0.1) and a two-sided α=0.05.
- 1:1 randomization to general adult ART services (Arm A) and MCH-focused ART services (Arm B)
- An expected proportion of women not retained in care and/or not virally suppressed at 12 months postpartum of 25% in the general adult ART services group (denoted πC), with this parameter ranging from 20-30% in different scenarios.
- The proportion of women retained in care and virally suppressed at 12 months postpartum in the MCH-focused ART services group (denoted πI) is expected to be 10%.
- Thus, the expected minimum difference in the combined endpoint of retained in care and virally suppressed between the MCH-focused and general adult ART strategies is 15% (i.e. πI - πC $\geq$ 0.15). This absolute difference corresponds to risk ratio of approximately 0.4.

Estimated sample sizes required under different assumptions

Proportion of women retained in care and VS at 12m postpartum in MCH group	Absolute increase in outcome, control minus intervention	Proportion of women retained in care and VS at 12m in general ART group	Sample size required (total)
0.1	0.1	0.2	572
0.15	0.1	0.25	708
0.2	0.1	0.3	824
**0.1**	**0.15**	**0.25**	**292**
0.15	0.15	0.3	348
0.2	0.15	0.35	396
0.1	0.2	0.3	184
0.15	0.2	0.35	214
0.2	0.2	0.4	238

This sample size has been adjusted to account for anticipated loss to follow-up before the primary outcome can be assessed at 12 months postpartum. We originally expected a maximum of 20% loss to follow up, however as the study has progressed we are experiencing higher rates of loss than originally projected. Accounting for this, we anticipate that up to390 women need to be enrolled into Phase 3 of the study, with approximately 195 randomized to each study group.

Note that from July 1 2013, local PMTCT guidelines shifted to recommend initiation of triple-drug ART for all HIV-infected pregnant women regardless of CD4 cell count. This policy shift has significant implications for interpretation of the study results, as participants enrolled before July 1 2013 (with ART eligibility based on CD4 cell count <350 cells/µl) represent a relatively morbid subset of the total population of pregnant women who are eligible for ART under the new guidelines. To ensure operational relevance of the study towards local policy and services, we plan to recruit the above mentioned sample size (n=390) in women initiating ART under the new policy (after 1 July 2013). Participants enrolled before this time (approximately 300 participants in Phase 1, 100 participants in Phase 2, and 90 participants in Phase 3) will be treated as a separate study population to be included in secondary analyses. Thus for the total study period, we anticipate up to 480 women enrolled into Phase 3.

In order to enrol up to 480 women into Phase 3, we anticipate enrolling a maximum of 600 women in Phase 2, and a maximum of 1600 women into Phase 1.

<u>Data management</u>
Data management will take place at the site following procedures established for multiple previous studies conducted in Gugulethu. Data collected on paper forms will be entered into a custom designed Microsoft Access database, maintained in a firewall-protected UCT server with nightly backups. The study database will be password-protected following standard password safety procedures. The database will be designed and maintained by a senior data manager who will develop the data dictionary, direct queries, and data quality assurance / quality control activities, and will supervise the data entry clerk. Data quality assurance will be in the form of robust database structure and platform, “front-end” data checks, including real-time database queries. Quality control will be through data checking scripts to identify out-of-range values, logic violations, and missing observations. Data editing will be based on reference to the form and/or source document in question; all data queries and responses will be logged, and edits will be implemented through separate program files. All study records will contain anonymous participant identification numbers, and no participant names or identifiers will be recorded.

Qualitative data from in-depth interviews will be translated and transcribed without participant names. All electronic files will be encrypted and password protected.

<u>Data analysis</u>
Data will be exported to Stata Version 12.0 (Stata Corporation, College Station, Texas) or R (Gnu Project) for analysis. All analyses will be carried out under supervision by the study statistician. All outcomes will be blinded to investigators until the data are cleaned and data analyses completed.

As described above, the primary analysis population (up to n=390) is all women enrolled into Phase 3 of the study who initiated ART during pregnancy after 1 July 2013 when the new provincial PMTCT guidelines were implemented. The subset of women in Phase 3 who initiated ART before this time (n=90, approximately) will contribute to a secondary analysis population comprised all women initiating ART in pregnancy and enrolled into Phase 3 during the study period (up to n=480).

For the primary objective, we hypothesize that women in the MCH-focused ART group will be more likely to <u>be retained in care</u> (defined as attending ART clinical appointments and/or medication refill visits in the window between 9 and 12 months postpartum) and <u>maintain viral suppression</u> (defined as <50 copies/mL, with secondary analyses at other thresholds) at 12 months postpartum, compared to women randomized to the general adult ART group. Comparisons of study groups at enrolment will be stratified by duration of ART during antenatal period (i.e., prior to randomization), and will examine demographic and HIV-related variables. Comparison of outcomes will be based on Fisher's exact test, comparing the proportion of women in each arm reaching the primary endpoint and its components; all statistical tests for the primary analysis will be two-sided at $\alpha=0.05$. Analyses will be by intention-to-treat, with secondary analyses stratified by duration of postnatal follow-up in the MCH-ART arm. The duration of viral suppression during the postpartum period will be defined as cumulative study time with a documented VL<50 copies/mL (with sub-analyses for <400 and <1000 copies/mL). Kaplan-Meier methods stratified by study group will be used to describe time to detectable viraemia and time to defaulting care, with proportional hazards models used to examine the independent influence of participant characteristics. Note that primary analyses will draw only on women enrolled into Phase 1 and initiating ART after 1 July 2013; women initiating ART before this time will be included as a secondary analysis population for selected analyses.

To address secondary objectives 2.1 and 2.2, data analysis of routinely collected measures of participants' use of PMTCT and ART services collected as part of Phases 1 and 2 will focus on the description of proportions of women receiving specific PMTCT services, and the delays calculated between various steps in the PMTCT cascade, including CD4 enumeration, return of CD4 results to patients and referral for ART, and initiation of ART. These analyses will employ proportions with exact 95% confidence intervals, and median delays (in days) with interquartile ranges. We will compare the proportions of women completing steps in the ART cascade, and the delays to specific steps, by basic demographic, psychosocial and clinical characteristics, collected during Phases 1 and 2. Comparisons will employ exact tests or rank-sum tests, as appropriate; statistical tests will be 2-sided at $\alpha=0.05$.

To address secondary objective 2.3, we will compare the two strategies for delivering ART during the postpartum period within subgroups of patients based on demographic, clinical and psychosocial characteristics measured antenatally (during Phase 2) and/or postnatally (during Phase 3). *A priori* subgroups will include:

- *Demographic characteristics* (age; gravidity/parity; education)
- *Clinical history & characteristics* (timing of HIV diagnosis; prior PMTCT or ART exposure)

- *Psychosocial characteristics* (measures of mental health and substance abuse; HIV- and ART-related knowledge and beliefs; adherence self-efficacy; perceived social support; disclosure of HIV status and ART use)
- *Antenatal ART use* (including duration of ART before delivery; adherence to ART during antenatal period)

Note that we will not adjust α for multiplicity but will name these as *a priori* subgroup analyses that are secondary to the primary comparison of interest (in the overall intention-to-treat population).

Secondary objective 2.4 will be addressed through analyses of the association between demographic, psychosocial and clinical measures (including characteristics of antenatal ART use) on the primary outcome. Analyses will parallel the pure count and time-to-event methods described for the primary objective. In addition, we will use proportional hazards models to identify measures independently associated with the primary outcome and its constituents; variable selection will be based on directed acyclic graphs developed *a priori* to help distinguish causal intermediates from confounding variables (for instance, antenatal ART adherence may be a causal intermediate through which demographic or psychosocial characteristics influence the primary outcome, rather than a confounder). All regression analyses will include trial arm as a covariate; other aspects of model building and diagnostics will follow standard approaches. Outputs will be expressed as hazard ratios and 95% confidence intervals.

Comparison of secondary outcomes between the two postnatal ART services will employ both pure count and person-time methods, as described above. Patterns of women's missed visits during the postpartum period (objective 2.5.A) will be analysed according to the frequency of different numbers of missed/late visits, and the timing of the missed/late visits during the postpartum. Breastfeeding practices (objective 2.5.B) will be analysed according to the duration of exclusive breastfeeding from birth; the duration of mixed feeding; and the timing of final infant exposure to breastmilk. Maternal viral suppression (objective 2.5.C) will be analysed according to the frequency and timing of detectable viraemia (with cutpoints at <50 and <1000 copies/mL) as well as 'survival' to detectable viraemia through 12 months. Incidence of infant respiratory and/or diarrhoeal illness (objective 2.5.D) will be analysed using person-time methods; comparisons of infant growth parameters will use dependent (intra-child) and independent (between child) comparisons. Rates of mother-to-child transmission will be compared at 6 weeks and 12 months using pure count methods (objective 2.5.E).

The BSID-III developmental scores by subscale, using raw, composite and percentile scores, will address objective 2.6. Developmental delay will be classified as more than 1 standard deviation from mean. Although the BSID-III normative scores are based on United States-based populations, similar scores were observed in a South African population, in a similar setting to ours[41]. Based on consultation with paediatric developmental specialists in our area, a difference in mean score of >5 will be used to define a clinically significant difference between subgroups. The primary exposure variable will be maternal HIV disease severity during pregnancy, as measured by clinical, immunological and virological measures at booking and delivery. Third variables will be identified *a priori* via directed acyclic graphs; these will include baseline, cumulative and time-varying measures (including maternal mental health, antenatal alcohol

and/or substance abuse, food insecurity, household asset index, gestational age, birth weight and subsequent infant growth). Throughout, units of analysis will be mother-infant pairs, with maternal and infant exposures as predictors of infant neurodevelopment. The distributional attributes of the BSID-III scores will determine the final generalized linear models of choice. A mixed effects model will be utilized to allow for repeated exposure measures and missing data.

Qualitative Data

Qualitative data on intervention acceptability in the context of women's ART initiation and follow-up will be used to address objective 2.5.E. The in-depth interview transcripts will be reviewed to develop a set of themes emerging from the interviews, with open-coding using HyperRESEARCH 2.8.3. (ResearchWare, Inc. 2009, http://www.researchware.com/) to allow new themes to emerge over time. In this way, the initial codes will be revised and refined to create a code-book to help guide further analysis. This codebook will be developed after 5-6 interviews are completed, and the codebook revisited after the full cohort of interviews has been transcribed. Thematic analysis will focus on dominant themes relayed by interview subjects, as well as the context in which themes emerged. Participant observation will be similarly coded with the same themes as in-depth interviews.

The qualitative analysis will draw on corresponding participant data from the quantitative arm of the study, and make use of this information to strengthen the analysis. At two points in the recruitment process, qualitative and quantitative participants will be compared according to key demographic indicators and adjusted accordingly to roughly resemble the quantitative arm.

Cost-effectiveness

Cost-effectiveness analysis (objective 2.5.F) will be analyzed from the health systems perspective. Costs will be defined as the mean cost per woman from the start of ANC through different points in time (eg, 6 or 12 months postpartum). Calculations will follow standard methods, with costs as quantities of resources utilized multiplied by unit costs. Resources include health services visits, laboratory investigations, and antiretroviral costs as well as the program-level costs (eg, patient education and adherence support materials) for each study group. Unit costs will be defined as the full economic cost for each item. Human resource costs will be calculated separately to enable an estimation of the number of different cadres of staff needed to provide services in each study group at different levels of scale. Outcome data will be used to calculate the incremental cost required to improve patient outcomes. Costs and outcomes will be discounted appropriately.

11. Ethical considerations

Ethical review

The study protocol, informed consent forms, all data collection tools, and other requested documents will be reviewed and approved by the Columbia University Medical Center Institutional Review Board (CUMC-IRB) and the University of Cape Town Faculty of Health Services Research Ethics Committee (UCT-REC). Subsequent to the initial review and approval, the CUMC-IRB and UCT-REC will review progress of the study at least annually.

Informed consent

Informed consent before enrolment into Phase 1, 2, or 3 will be delivered in participants' home language (isiXhosa) by trained interviewers following a standardized script. This script details the purpose of the study, the nature of randomization, study procedures throughout the antenatal and postnatal periods, and the risks and benefits to mothers and infants that participants may encounter during the study. Here and throughout the study, study staff will emphasize to participants that:

- Participation is entirely voluntary, and their choice regarding participation will in no way influence the quality of antenatal or postnatal routine medical care for mothers or their infants
- Women may exit the study at any time for any reason without compromising the quality of health care received.

Note that separate informed consent documents will be used in this study:

1. Informed Consent #1: This consent will be completed by all HIV-infected pregnant women making their first antenatal visit who meet eligibility criteria for inclusion in Phase 1 (up to 1000 women during the 12 month recruitment period). This consent form provides details of the overall purpose of the study and outlines the following study procedures: (1) completion of an enrolment questionnaire (including demographic and behavioural questions); (2) blood draw, and; (3) permission to conduct chart review and data abstraction from routine clinical records through pregnancy and post-partum period; (4) permission to contact participant regarding future research.

2. Informed Consent #2: This consent will be completed by a subset of women from Phase 1 who are deemed ART-eligible and who meet eligibility criteria for inclusion in Phase 2. This consent form introduces the overall purpose and process of the remainder of the study (Phase 2/3) and the specific procedures for Phase 2 antenatal/postnatal measurement visits (through 1 week postpartum).

3. Informed Consent #3: This consent will be completed by a subset of women from Phase 2 who initiated ART during the antenatal period, who are breastfeeding their infants, and who meet other eligibility criteria for inclusion in Phase 3. This consent form details the overall purpose of the study, the two study arms, the concept and process of randomization, and the procedures for Phase 3 postnatal measurement visits (through 18 months postpartum).

4. Informed Consent for Infant Neurodevelopmental Assessment: This consent will be completed by Phase 3 participants who complete the 12 months postpartum visit and who have infants who are HIV-uninfected and meet other eligibility criteria for participation in this additional assessment. This consent form details the overall purpose of the assessment and the procedures for conducting the developmental assessment.

English versions of the informed consent documents are provided in the Appendix to this document. Translated isiXhosa versions (as well as a certification of their translation and back-translation) will be lodged with the IRB/REC before the start of the study.

Risks
The potential risks to participants in the study include:

- Risks associated with collection of biologic specimens, through complications of phlebotomy.
- Risks associated with collection of self-reported behavioural and psychosocial information, related to psychosocial distress raised by questionnaire items involving social support, mental health, or disclosure of HIV status or ART use.
- Risks due to loss of confidentiality due to study procedures—for instance, in the process of data collection or participant follow-up.

All participants will be informed of these risks, and the strategies to minimize these, as part of the informed consent process. The alternative to participation is the referred initiation strategy, and each of these classes of risk are encountered during routine health care services. However, the study team has integrated planned specific steps in study design and conduct to minimize the possibility of these risks. These steps draw directly from prior experiences conducting research on HIV prevention and treatment in Gugulethu and similar communities across Cape Town.

Benefits

Direct benefit
The major potential direct benefit from participating in this study is optimized ART use during pregnancy and postpartum. This, in turn, is likely to minimize the risk of mother-to-child HIV transmission while maximizing maternal health (recognizing that optimizing the health of an HIV-infected mother has benefits both for her and her children, whether HIV infected, HIV-exposed, or HIV-uninfected). Given the high risk of MTCT and high risk of morbidity and mortality in ART eligible pregnant women, these benefits substantially outweigh any risks associated with participation in the study.

Indirect benefit
By identifying the optimal strategy for delivering ART to women during the postpartum period, this study has the potential to lead to improved ART services for HIV-infected women in Cape Town, the Western Cape Province, and across South Africa. To this end, the involvement of provincial policy makers involved in HIV care and treatment and maternal and child health will help maximize the indirect benefits of the study through strengthened public sector health care services for HIV-infected pregnant women.

Compensation
Participants will be given up to R150, R20 in cash to cover the cost of transport to their next scheduled study measurement visit, R80 in the form of grocery vouchers and up to a maximum of R50 in refreshments, and a small gift at the first and last post-partum visit, for their time and effort in study measurement visits for Phase 2 and Phase 3. Note that no compensation or reimbursement in any form will be provided to women attending routine ANC and/or ART-related visits at any time during the study. The subset of women participating in the qualitative in-depth interviews will be given R100 in the form of grocery vouchers, cash to cover transport and refreshments for their time and effort at the end of each interview.

Confidentiality

The following steps will be taken to minimize the risk of any loss of confidentiality throughout study design and conduct.

- All personnel involved in data collection and management will undergo specific training for the study in confidentiality and related patient protection issues.
- Following standard practice, all patient- and study-related information will be kept in locked cabinets at either the study office in Gugulethu or at UCT.
- Anonymous participant identification numbers will be used on all study documents. Collection of participant names and other identifiers will be restricted to informed consent documents, patient tracing materials, and a study identification key, all of which will be kept in a locked cabinet in the study office at Gugulethu and at UCT separate from other study documentation and accessible only by the project coordinator and local PI. No CRF will include participant name, including CRF that may reflect HIV status of women or their children (including HIV test results or information about ART use).
- All electronic records will be kept in password-protected files. All electronic communications of study data will be through password-protected, encrypted files. All data storage at the University of Cape Town will be within a firewall-protected SQL server.

While efforts will be made to minimize the loss of confidentiality, in the event that staff learn that the participant is a threat to themselves or to others or of possible child abuse or neglect, the proper authorities will be notified. This exception will be included in all study informed consent forms.

Internal monitoring

Throughout the conduct of the study, internal study monitoring will be led by the study PIs. During the first six months of the study, study PIs, co-investigators and the study coordinator will participate in weekly conference calls to monitor the rate of participant enrolment and the integrity of protocol implementation (including the completion of informed consent and quality of study measures). Thereafter, the internal monitoring process will become biweekly, with conference calls to review the above features as well as participant retention and safety endpoints.

Patient clinical care

In addition to these internal reviews, several SOPs will be used in the study to optimize participant safety:

- Any woman found to have defaulted ART during any Phase of the study will be re-referred to the relevant ART service (general adult or MCH-focused) via the Gugulethu MOU.
- Any woman found to be non-adherent to ART at any study measurement visit will receive intensive counselling on ART use, and referred to the relevant ART service for follow-up.
- Any woman found to be experiencing domestic or partner violence in any form will be referred to the main local NGO supporting victims of domestic violence (MOSAIC) and the South African Police Service (per the Domestic Violence Act, No. 116 of 1998) with follow-up to ensure adequate attention.

- Any child found to be HIV-infected will be referred immediately to the nearest paediatric ART service for assessment and infant ART initiation.
- If any infant health care needs identified, the infant will be referred to the appropriate paediatric health services.

Use of Information and Publications

Publication or presentation of the results of this study will be agreed upon in collaboration with the study investigators. Note that the funding agency has no input in the decision to present or publish study data or the nature of the data that are presented or published.

Appendices

Appendix A: Informed Consent Form, Phase 1
Appendix B: Informed Consent Form, Phase 2
Appendix C: Informed Consent Form, Phase 3
Appendix D: Data Collection Tools
Appendix E: Informed Consent Form, Qualitative Interviews
Appendix F: Qualitative Interview Guide
Appendix G: Table of Assessments

References

1. Mofenson LM. Prevention in neglected subpopulations: prevention of mother-to-child transmission of HIV infection. *Clin Infect Dis* 2010,**50 Suppl 3**:S130-148.
2. UNICEF. *Children and AIDS: Fifth Stocktaking Report, 2010*. Geneva: UNICEF, UNAIDS, WHO, UNFPA and UNESCO 2010.
3. Townsend CL, Cortina-Borja M, Peckham CS, de Ruiter A, Lyall H, Tookey PA. Low rates of mother-to-child transmission of HIV following effective pregnancy interventions in the United Kingdom and Ireland, 2000–2006. *AIDS* 2008,**22**:973-981.
4. Mofenson LM. Protecting the Next Generation — Eliminating Perinatal HIV-1 Infection. *New Engl J Med* 2010,**24**:2316-2318.
5. UNAIDS. *Global report: UNAIDS report on the global AIDS epidemic, 2010*. Geneva: UNAIDS: UNAIDS/10.11E | JC1958E; 2010.
6. Goga A, Dinh TH, Jackson D, for the SAPMTCTE study group. *Evaluation of the Effectiveness of the National Prevention of Mother-to-Child Transmission (PMTCT) Programme on Infant HIV measured at Six Weeks Postpartum in South Africa, 2010.* Pretoria: South African Medical Research Council, National Department of Health of South Africa and PEPFAR/US Centers for Disease Control and Prevention.; 2012.
7. Dinh TH, Goga A, Jackson D. Impact of South Africa's PMTCT Programs on Perinatal HIV Transmission: Results of the 1st Year Implementing 2010 WHO Recommended Guidelines. In: *4th International workshop on Pediatric HIV* Washington DC; 2012.
8. National Department of Health. *Strategic Plan for Maternal, Newborn, Child and Women's Health (MNCWH) and Nutrition in South Africa, 2012-2016.* Pretoria: NDOH; 2012.
9. National Department of Health. *Operational Plan for Accelerating Scale Up and Improvement of the Quality of Services for Prevention of Mother to Child Transmission (PMTCT) in the Context of Integrated Maternal and Child Health Care in South Africa.* Pretoria: NDOH; 2009.
10. Bekker LG, Black V, Myer L, Rees H, Cooper D, Mall S*, et al.* Guideline on safer conception in fertile HIV-infected individuals and couples. *S Afr J HIV Med* 2011,**12**:31-44.
11. Hoffman RM, Black V, Technau K, Van Der Merwe KJ, Currier J, Coovadia A*, et al.* Effects of Highly Active Antiretroviral Therapy Duration and Regimen on Risk for Mother-to-Child Transmission of HIV in Johannesburg, South Africa. *J Acquir Immune Defic Syndr* 2010,**54**:35-41.
12. Ferguson L, Grant AD, Watson-Jones D, Kahawita T, Ong'ech JO, Ross DA. Linking women who test HIV-positive in pregnancy-related services to long-term HIV care and treatment services: a systematic review. *Trop Med Int Health* 2012.
13. Braun M, Kabue MM, McCollum ED, Ahmed S, Kim M, Aertker L*, et al.* Inadequate coordination of maternal and infant HIV services detrimentally affects early infant diagnosis outcomes in Lilongwe, Malawi. *J Acquir Immune Defic Syndr* 2011,**56**:e122-128.
14. Nachega JB, Marconi VC, van Zyl GU, Gardner EM, Preiser W, Hong SY*, et al.* HIV treatment adherence, drug resistance, virologic failure: evolving concepts. *Infect Disord Drug Targets* 2011,**11**:167-174.

15. Nachega JB, Uthman OA, Anderson J, Peltzer K, Wampold S, Cotton M, *et al.* Adherence to antiretroviral therapy during and after pregnancy in low-, middle- and high-income countries: a systematic review and meta-analysis. *AIDS* 2012,**in press**.
16. Boyles TH, Wilkinson LS, Leisegang R, Maartens G. Factors influencing retention in care after starting antiretroviral therapy in a rural South African programme. *PLoS ONE* 2011,**6**:e19201.
17. Kaplan R, Orrell C, Zwane E, Bekker LG, Wood R. Loss to follow-up and mortality among pregnant women referred to a community clinic for antiretroviral treatment. *AIDS* 2008,**22**:1679-1681.
18. Van Cutsem G, Ford N, Hildebrand K, Goemaere E, Mathee S, Abrahams M, *et al.* Correcting for mortality among patients lost to follow up on antiretroviral therapy in South Africa: a cohort analysis. *PLoS One* 2011,**6**:e14684.
19. Wang B, Losina E, Stark R, Munro A, Walensky RP, Wilke M, *et al.* Loss to follow-up in a community clinic in South Africa--roles of gender, pregnancy and CD4 count. *S Afr Med J* 2011,**101**:253-257.
20. Kuhn L, Aldrovandi GM, Sinkala M, Kankasa C, Mwiya M, Thea DM. Potential impact of new WHO criteria for antiretroviral treatment for prevention of mother-to-child HIV transmission. *AIDS* 2010,**24**:1374-1377.
21. European Collaborative Study. Time to Undetectable Viral Load after Highly Active Antiretroviral Therapy Initiation among HIV-Infected Pregnant Women. *Clin Infect Dis* 2007,**44**:1647-1656.
22. Fitzgerald FC, Bekker L-G, Kaplan R, Myer L, Lawn SD, Wood R. Mother-to-child transmission of HIV in a community-based antiretroviral clinic in South Africa. *S Afr Med J* 2010,**100**:827-831.
23. Shapiro RL, Hughes MD, Ogwu A, Kitch D, Lockman S, Moffat C, *et al.* Antiretroviral Regimens in Pregnancy and Breast-Feeding in Botswana. *New Engl J Med* 2010,**362**:2282-2294.
24. Nachega JB, Mills EJ, Schechter M. Antiretroviral therapy adherence and retention in care in middle-income and low-income countries: current status of knowledge and research priorities. *Curr Opin HIV AIDS* 2010,**5**:70-77.
25. Thompson MA, Mugavero MJ, Amico KR, Cargill VA, Chang LW, Gross R, *et al.* Guidelines for improving entry into and retention in care and antiretroviral adherence for persons with HIV: evidence-based recommendations from an International Association of Physicians in AIDS Care panel. *Ann Intern Med* 2012,**156**:817-833, W-284, W-285, W-286, W-287, W-288, W-289, W-290, W-291, W-292, W-293, W-294.
26. Cornell M, Grimsrud A, Fairall L, Fox MP, van Cutsem G, Giddy J, *et al.* Temporal changes in programme outcomes among adult patients initiating antiretroviral therapy across South Africa, 2002-2007. *AIDS* 2010,**24**:2263-2270.
27. Myer L, Cornell M, Fox MP, Garone D, Giddy J, Prozesky H, *et al.* Loss to Follow-up and Mortality among Pregnant and Non-pregnant Women Initiating ART: South Africa. In: *CROI 2012*. Seattle, Washington, USA; 2012.
28. National Department of Health. *Saving mothers, 2008-2010. Firth report on the confidential enquriries into maternal deaths in South Africa.* Pretoria: NDOH; 2012.
29. Coutsoudis A, England K, Rollins N, Coovadia H, Newell M-L, Bland R. Women' s morbidity and mortality in the first 2 years after delivery according to HIV status. *AIDS* 2010,**24**:2859-2866.

30. Palombi L, Marazzi MC, Voetberg A, Magid NA. Treatment acceleration program and the experience of the DREAM program in prevention of mother-to-child transmission of HIV. *AIDS* 2007,**21 Suppl 4**:S65-71.
31. The Kesha Bora Study Group. Eighteen-Month Follow-Up of HIV-1 – Infected Mothers and Their Children Enrolled in the Kesho Bora Study Observational Cohorts. *J Acquir Immune Defic Syndr* 2010,**00**:1-9.
32. Doherty T, Sanders D, Jackson D, Swanevelder S, Lombard C, Zembe W, *et al.* Early cessation of breastfeeding amongst women in South Africa: an area needing urgent attention to improve child health. *BMC Pediatr* 2012,**12**:105.
33. Goga AE, Doherty T, Jackson DJ, Sanders D, Colvin M, Chopra M, *et al.* Infant feeding practices at routine PMTCT sites, South Africa: results of a prospective observational study amongst HIV exposed and unexposed infants - birth to 9 months. *Int Breastfeed J* 2012,**7**:4.
34. Doherty T, Chopra M, Tomlinson M, Oliphant N, Nsibande D, Mason J. Moving from vertical to integrated child health programmes: experiences from a multi-country assessment of the Child Health Days approach in Africa. *Trop Med Int Health* 2010,**15**:296-305.
35. Stinson K, Boulle A, Coetzee D, Abrams EJ, Myer L. Initiation of highly active antiretroviral therapy among pregnant women in Cape Town, South Africa. *Trop Med Int Health* 2010,**15**:825-832.
36. Myer L, Zulliger R, Bekker LG, Abrams E. Systemic delays in the initiation of antiretroviral therapy during pregnancy do not improve outcomes of HIV-positive mothers: a cohort study *BMC Pregnancy & Childbirth* in press.
37. Stinson K, Myer L. Barriers to initiating antiretroviral therapy during pregnancy: a qualitative study of women attending services in Cape Town, South Africa. *African Journal of AIDS Research* 2012,**11**:65-73.
38. Mepham S, Zondi Z, Mbuyazi A, Mkhwanazi N, Newell ML. Challenges in PMTCT antiretroviral adherence in northern KwaZulu-Natal, South Africa. *AIDS Care* 2011,**23**:741-747.
39. Mnyani CN, McIntyre JA, Myer L. The reliability of point-of-care CD4 testing in identifying HIV-infected pregnant women eligible for antiretroviral therapy. *J Acquir Immune Defic Syndr* 2012,**60**:260-264.
40. Myer L, Zulliger R, Black S, Pienaar D, Bekker LG. Pilot programme for the rapid initiation of antiretroviral therapy in pregnancy in Cape Town, South Africa. *AIDS Care* 2012,**24**:986-992.
41. Rademeyer V, Jacklin L. A study to evaluate the performance of black South African urban infants on the Bayley Scales of Infant Development III. Vol 72013.

Appendix G: Table of Assessments

Assessment Instrument		Description	Adapted from:
1.	Demographics & Medical History (Baseline and Follow-up)	Demographics, employment, housing, clinic transportation, household composition, parity, current partnerships, baseline medical characteristics (Tb history, hospitalizations), HIV testing and disclosure history, previous use of ART (PMTCT/other ART use), pregnancy outcomes	In House
2.	HIV/AIDS Knowledge Inventory	10-item inventory assess knowledge about how HIV is transmitted in general as well as specific questions about mother-to child-transmission of HIV	Kalichman, S. C., & Simbayi, L. C. (2003). HIV testing attitudes, AIDS stigma, and voluntary HIV counselling and testing in a black township in Cape Town, South Africa. *Sex Transm Infect*, 79(6), 442-447.
3.	HIV Treatment Knowledge Inventory	8-item questionnaire asking participants about their understanding of how ARVs work for both mothers and their infants.	Wagner, G.J., Remien, R.H., Dolezal, C., Carballo-Dieguez, A. (2002). Correlates of adherence to combination antiretroviral therapy among HIV+ members of HIV mixed status couples. *AIDS Care*, 14(1): 105-109.<br><br>Wagner, G.J., Kanouse, D.E., Koegel, P., Sullivan, G. (2004) Correlates of HIV antiretroviral adherence in persons with serious mental illness. *AIDS Care*, 16(4): 501-506.
4.	Beliefs about Medications Questionnaire	7-item antiretroviral version of the BMQ that assesses participant beliefs regarding the use of medication to treat their HIV during pregnancy and postpartum	Horne, R., Weinman, J., & Hankins, M. 1999. The beliefs about medicines questionnaire: The development and evaluation of a new method for assessing the cognitive representation of medication. *Psychology and Health*, 14, 1-24.
5.	Adherence Self-Efficacy Scale	15-item scale measuring self-efficacy and confidence for medication and medical care adherence in general and specifically during pregnancy and postpartum period. Respondents rate items regarding specific components of medical care management on a	Chesney, M.A., Ickovics, J.R., Chambers, D.B., Giffrord, A.L., Nedig, J., Zwickl, B. and Wu, A.W. (2000) Self-reported adherence to antiretroviral medications among participants in HIV clinical trials: the AACTG adherence instruments. *AIDS*

		scale from 1 (not at all confident) to 5 (completely confident)	*Care*, 12, 255–266.
6.	Kessler 10 (K10)	10-item scale regarding the degree to which situations in one's life during the last month were appraised as stressful	Kessler, R. C., Andrews, G., Colpe, L. J., Hiripi, E., Mroczek, D. K., & Normand, S. T., et al. (2002). Short screening scales to monitor population prevalences and trends in nonspecific psychological distress. *Psychological Methods*, 32, 959-976.<br><br>Kessler, R. C., Barker, P. R., Colpe, L. J., Epstein, J. F.,<br>Gfroerer, J. C., & Hiripi, E., et al. (2003). Screening for serious mental illness in the general population. *Archives of General Psychiatry*, 60(2), 184-189.
7.	EPDS	10-item scale used for screening of depression during and after pregnancy.	Cox, JL, et al. Detection of postnatal depression: development of the 10-item Edinburgh Postnatal Depression Scale. *Br J Psychiatry*. 1987;150:782-786.
8.	AUDIT	10-item scale used for screening of alcohol use and alcohol-related problems	Saunders, J.B, Aasland, O.G., Babor, T.F., De La Fuente J.R., Grant, M. (1993), Development of the Alcohol Use Disorders Identification Test (AUDIT): WHO Collaborative Project on Early Detection of Persons with Harmful Alcohol Consumption-II. *Addiction*, 88: 791–804.
9.	DUDIT	11-item scale used for screening of drug use and drug-related problems	Berman AH, Bergman H, Palmstierna T, Schlyter F: Evaluation of the Drug Use Disorders Identification Test (DUDIT) in Criminal Justice and Detoxification Settings and in a Swedish Population Sample**.** *Eur Addict Res* 2005;11:22-31
10.	Violence Against Women Assessment	12-item questionnaire used to assess the prevalence, frequency and severity of different forms of violence by intimate partners	WHO Violence Against Women Assessment
11.	Social Impact Scale	7-item Likert-scale instrument assessing perception of illness related stigma.	Fife, B. L., & Wright, E. R. (2000). The dimensionality of stigma: a comparison of its impact on the self of persons with HIV/AIDS and cancer. *J Health Soc Behav*, 41(1),

			50-67.
12.	Perceived Availability of Support	12-item scale regarding instrumental ways in which the participant perceives that others are available to assist with his/her HIV illness and health behaviour, specifically during and after pregnancy.	Cohen, S. Psychosocial models of the role of social support in the etiology of physical diseases. *Health Psychology*, 7(3), 269-297.
13.	Patient Provider Relationship Scale	19-item questionnaire that aims to quantify the patient-provider relationship and assess whether it has an impact on their receipt of HIV care during and after pregnancy.	Oliver M. Barry, Anne-Marie Bergh, Jennifer D. Makin, Elsie Etsane, Trace S. Kershaw & Brian W.C. Forsyth (2012): Development of a measure of the patient–provider relationship in antenatal care and its importance in PMTCT, *AIDS Care*: Psychological and Socio-medical Aspects of AIDS/HIV, 24:6, 680-686
14.	Infant Feeding Intentions/Practices	3 separate assessments asked at distinct time points- 1) during pregnancy; 2) 7-days postpartum; and 3) from 6-weeks pp thru 12 months pp. Questions aim to assess breast feeding intentions during pregnancy and actual postpartum practices.	Jackson D, Chopra M, Doherty T, Colvin M, Levin J, Willumsen J, Goga A & Moodley P. Operational effectiveness and 36 week HIV free survival in the South African programme to prevent mother-to-child transmission of HIV-1. *AIDS*, 21:509-516.
15.	Family Planning Use/Intentions	7-item questionnaire assessing previous use of family planning methods and use of family planning methods in the postpartum period.	In house
16.	London Measure of unplanned pregnancy	6-item questionnaire measuring pregnancy planning/intention	Barrett G, Smith SC, Wellings K.(2004) Conceptualisation, development and evaluation of a measure of unplanned pregnancy Journal of Epidemiology and Community Health **58**:426-433
17.	Maternal Adherence	2 separate assessments asked at baseline and all follow-up visits. Aims to assess medication side effects, adherence to medication and reasons for non-adherence.	Wilson I, Fowler F, Cosenza C, Rogers W, Michaud J, Bentkover J, Kogelman L, Rana A. Lessons from Cognitive Testing of Self-Report Adherence Items. Oral presentation, 7th International Conference on HIV Treatment and Prevention Adherence, Miami, FL. June 3-5, 2012.
18.	Infant Adherence	2 separate assessments asked at less than 7-days pp, 6-weeks pp and 3 months pp. Aims to assess medication side effects, adherence to medication	Wilson I, Fowler F, Cosenza C, Rogers W, Michaud J, Bentkover J, Kogelman L, Rana A. Lessons from Cognitive Testing of Self-Report

		and reasons for non-adherence.	Adherence Items. Oral presentation, 7th International Conference on HIV Treatment and Prevention Adherence, Miami, FL. June 3-5, 2012.
19.	Child Grants	8-item questionnaire assessing grant support received for newborn child.	Jackson D, Chopra M, Doherty T, Colvin M, Levin J, Willumsen J, Goga A & Moodley P. Operational effectiveness and 36 week HIV free survival in the South African programme to prevent mother-to-child transmission of HIV-1. *AIDS*, 21:509-516.
20.	Infant Anthropometry	Length, weight, head circumference and middle upper arm circumference	In house
21.	Infant Demographics & Medical History	Infant health and health care (Pneumonia, diarrhoea, TB, HIV)	In house
22.	Food security	10-item questionnaire assessing household food security	Labadorios D, Mchiza Z, Steyn N, Gericke G, Maunder E, Davids Y & Parker W (2011) Food security in South Africa: a Review if national surveys. Bull World Health Organ 2011;**89**:891–899<br>Swindale A & Bilinksy P (2006). Development of a universally applicable household food insecurity measurement tool: process, current status, and outstanding issues. J.Nutr. **136**:1449S-1452S
23.	Resource Interview	10-item questionnaire assessing perceptions of health and impact of health issues on daily activities	In house
24.	WHO standard verbal autopsy for investigating causes of death in infants and children	A standard tool to investigate cause of death	World Health Organization. A Standard Verbal Autopsy Method for Investigating Causes of Death in Infants and Children WHO/CDS/CSR/ISR/99.4. Geneva: WHO, 1999.
25.	Bayley Scales of Infant and Toddler Development 3rd edition (BSID-III)	A standard series of measurements used to assess the motor, language and cognitive development of infants and toddlers.	Bayley, N. The Bayley Scales of Infant and Toddler Development, 3rd edition.
